# Supplementary material for: Decoding living systems: Reassessing crop model frontiers via biological dynamics and optimized phenotype
Source: PLoS One. 2026 Mar 11;21(3):e0343530. doi: 10.1371/journal.pone.0343530 (PMC12978445; doi:10.1371/journal.pone.0343530)
Supplement: S1 Table — Bold indicates the highest sensitivity per output. (PDF) [file pone.0343530.s002.pdf]

Table S1. Relative Sensitivity Index (RSI) from 20 Morris method replications. Bold = highest sensitivity per output.

**Coefficient**	**Grain Yield**	**Biomass**	**Anthesis**	**Maturity**	**N° Grains**	**N° Tillers**
P1	0.46 ± 0.04	0.55 ± 0.04	**0.93 ± 0.02**	0.57 ± 0.19	0.46 ± 0.04	0.88 ± 0.02
P2O	0.42 ± 0.04	0.45 ± 0.04	0.79 ± 0.12	**0.64 ± 0.08**	0.43 ± 0.04	0.76 ± 0.11
P2R	0.37 ± 0.03	0.39 ± 0.03	0.00 ± 0.00	0.50 ± 0.05	0.37 ± 0.03	0.05 ± 0.03
P5	0.42 ± 0.04	0.42 ± 0.04	0.70 ± 0.05	0.35 ± 0.06	0.42 ± 0.04	0.66 ± 0.05
G1	0.45 ± 0.04	0.06 ± 0.03	0.00 ± 0.00	0.00 ± 0.00	0.46 ± 0.04	0.00 ± 0.00
G2	0.44 ± 0.04	0.05 ± 0.02	0.00 ± 0.00	0.00 ± 0.00	**0.64 ± 0.03**	0.00 ± 0.00
G3	**0.59 ± 0.04**	0.48 ± 0.05	0.00 ± 0.00	0.00 ± 0.00	0.59 ± 0.04	**0.93 ± 0.01**
PHINT	0.42 ± 0.02	**0.70 ± 0.02**	0.86 ± 0.02	0.47 ± 0.05	0.42 ± 0.02	0.84 ± 0.03
THOT	0.45 ± 0.06	0.05 ± 0.02	0.00 ± 0.00	0.00 ± 0.00	0.45 ± 0.06	0.00 ± 0.00
TCLDP	0.00 ± 0.00	0.00 ± 0.00	0.00 ± 0.00	0.00 ± 0.00	0.00 ± 0.00	0.00 ± 0.00
TCLDF	0.00 ± 0.00	0.00 ± 0.00	0.00 ± 0.00	0.00 ± 0.00	0.00 ± 0.00	0.00 ± 0.00
