# Supplementary material for: Decoding living systems: Reassessing crop model frontiers via biological dynamics and optimized phenotype
Source: PLoS One. 2026 Mar 11;21(3):e0343530. doi: 10.1371/journal.pone.0343530 (PMC12978445; doi:10.1371/journal.pone.0343530)
Supplement: S2 Table — Statistical significance: *** p<0.001, ** p<0.01, * p<0.05, ns p≥0.05. Correlations with R2<0.10 are considered practically negligible despite statistical significance due to large sample sizes (n>1,300 per environment). (PDF) [file pone.0343530.s003.pdf]

Table S2. Pearson correlation (R^2) between HI-WUE index and phenotypic outputs across four environments. Statistical significance: *** $p < 0.001$, ** $p < 0.01$, * $p < 0.05$, ns $p \geq 0.05$. Correlations with $R^2 < 0.10$ are considered practically negligible despite statistical significance due to large sample sizes ($n > 1{,}300$ per environment).

**Env**	**Metric**	**GY**	**Biomass**	**HI**	**N°Grains**	**LAI**	**WUE**	**Root**	**Anthesis**	**Maturity**
1	R^2	0.78	0.04	**0.88**	0.39	0.01	**0.86**	0.02	0.00	0.01
	p-value	***	***	***	***	ns	***	**	ns	*
2	R^2	0.86	0.03	**0.97**	0.56	0.05	**0.93**	0.02	0.06	0.08
	p-value	***	***	***	***	***	***	**	***	***
3	R^2	0.78	0.00	**0.95**	0.60	0.00	**0.88**	0.00	0.00	0.00
	p-value	***	ns	***	***	ns	***	ns	ns	ns
4	R^2	0.85	0.02	**0.97**	0.50	0.03	**0.93**	0.01	0.04	0.05
	p-value	***	**	***	***	***	***	*	***	***
