# Supplementary material for: Decoding living systems: Reassessing crop model frontiers via biological dynamics and optimized phenotype
Source: PLoS One. 2026 Mar 11;21(3):e0343530. doi: 10.1371/journal.pone.0343530 (PMC12978445; doi:10.1371/journal.pone.0343530)
Supplement: S3 Table — Env1: high water retention, high precipitation (30% area); Env2: medium retention, low precipitation (18%); Env3: medium retention, high precipitation (21%); Env4: low retention, low precipitation (20%). Values represent the best individual identified across 40 generations of genetic algorithm optimization. (PDF) [file pone.0343530.s004.pdf]

Table S3. Optimal genetic coefficients and predicted crop performance for each environment. Env 1: high water retention, high precipitation (30% area); Env 2: medium retention, low precipitation (18%); Env 3: medium retention, high precipitation (21%); Env 4: low retention, low precipitation (20%). Values represent the best individual identified across 40 generations of genetic algorithm optimization.

**Parameter**	**Range**	**Env 1**	**Env 2**	**Env 3**	**Env 4**	**Description**
Genetic coefficients						
P1 (GDD)	150–800	526.6	605.0	538.1	605.0	Vegetative phase thermal time
P5 (GDD)	150–850	372.1	207.0	247.8	207.0	Grain filling duration
P2R (GDD)	5–300	237.4	112.8	204.6	112.8	Photoperiod sensitivity
PHINT (GDD)	55–90	73.9	72.0	74.1	72.0	Phyllochron interval
P2O (h)	11–13	11.7	11.7	12.1	11.7	Critical photoperiod
G1 (#/g)	38–540	64.0	61.0	61.0	61.0	Spikelet number coefficient
G2 (g)	0.015–0.030	0.025	0.025	0.027	0.025	Single grain weight
G3	0.7–1.97	0.85	0.97	0.95	0.97	Tillering coefficient
Predicted performance						
Grain yield (kg/ha)		**4,837**	4,014	4,213	3,743	
Biomass (kg/ha)		8,895	7,200	7,214	6,802	
HI		0.54	0.56	**0.58**	0.55	
WUE ($kg\ ha^{-1} mm^{-1}$)		**6.17**	5.97	5.84	5.97	
Grain number (/m^2)		19,350	16,056	15,602	14,971	
Tiller number (/m^2)		956	1,253	906	1,242	
Anthesis (days)		87	80	80	80	
Maturity (days)		116	101	103	101	
LAI (m^2/m^2)		3.73	3.23	2.97	3.03	
Optimization metrics						
Best fitness (HI-WUE)		0.955	0.955	**0.973**	0.948	
Convergence (gen)		23	20	10	20	Generation at 95% max fitness
