# Supplementary material for: Decoding living systems: Reassessing crop model frontiers via biological dynamics and optimized phenotype
Source: PLoS One. 2026 Mar 11;21(3):e0343530. doi: 10.1371/journal.pone.0343530 (PMC12978445; doi:10.1371/journal.pone.0343530)
Supplement: Attachment — Submitted filename: Letter to the Author - PONE-D-25-26754.pdf [file pone.0343530.s006.pdf]

Dear Author,

Thank you for submitting your work to PLOS ONE. Below I provide a review of your manuscript.

PLOS ONE evaluates **technical soundness, clarity, ethics/data availability, and reproducibility** rather than perceived impact; my notes, analysis and comments are organized accordingly and include precise locations so you can revise efficiently.

Summary of the study (what the paper does)

You present an integrated framework that couples the **CERES-Rice** mechanistic crop model with a **Genetic Algorithm (GA)** and a **Morris global sensitivity analysis** to propose a rice **ideotype** that jointly improves **harvest index** and **water-use efficiency (WUE)** across contrasting environments in Senegal. The workflow: define decision-variable bounds (CERES genetic parameters), run Morris to identify influential factors, optimize a composite objective (**HI-WUE**) with a GA, and then compare the optimized "virtual cultivar" to 21 field-characterized cultivars with multiple **similarity metrics**. Data and code availability are noted, with simulations and processing scripts referenced.

Manuscript metadata

- **Title:** *Mechanistic crop modelling and AI for ideotype optimization: Crop-scale advances to enhance yield and water use efficiency* (p.1).
- **Authors:** The draft clearly states that the only author is **Edgar S. Correa**, MEng, PhD candidate at the School of Engineering of the Pontificia Universidad Javeriana, Bogota, Colombia, The CIRAD, INRAE, Institut Agro, Montpellier, France and the CIRAD, UMR AGAP Institut, F-34398, Montpellier, France.
- **Length: 26 pages**.
- **Figures: 10** figures (Fig. 1–10).
- **Tables: 1** table (Table 1).
- **Equations:** At least **7** numbered equations (Eq. (1)–(6) explicitly, plus a numbered display "(7)" for *Normalized Similarity* on p.11, l.25).
- **Algorithms :** 3 algorithms.
- **References: 68** entries in the References section (pp.21–26).

(Counts computed from the uploaded PDF **PONE-D-25-26754_reviewer.pdf**.)

0) Project & Environment Setup (Reproducibility Gate)

• Fix DSSAT/CERES versions, OS, seeds; container or env.yaml
• Repo layout: /data, /models, /code, /config, /results, /docs
• Artifact: env.yaml, project.yaml, README

v

1) Inputs & Calibration (Data Gate)

• Weather (POWER/ground), soils (profiles), management
• Calibrate observed cultivars' genetic params (P1,P2O,P2R,P5,PHINT,G1–G3)
• Artifact: calib_cultivars.csv; curated weather/soil/management files

v

2) Morris Global Sensitivity

• Specify k, p, Δ, r; outputs: HI, WUE, phenology
• Run CERES per sample across environments; compute μ* and σ
• Artifacts: morris_design.csv, morris_effects.csv, bounds.yaml

v

3) Objective Definition

Option A: $J = HI + \alpha \cdot WUE$ (scale/justify α)
Option B: Multi-objective (Pareto: HI, WUE)
• Artifact: objective.yaml (scaling, α or Pareto)

v

4) GA Ideotype Optimization (Across Environments)

• Init pop via LHS; constraints (phenology windows)
• Evaluate: CERES → HI,WUE → aggregate; penalties if violated
• Selection/crossover/mutation, elitism; multi-seed runs
• Artifacts: ga_runs/*, best_theta.csv, convergence traces

v

5) Post-Optimization Simulation & Diagnostics

• Simulate θ* across sites/years; compute HI, WUE, ET, yield, phenology
• Ablations (±10% params) and uncertainty (bootstrap/weather MC)
• Artifacts: ideotype_performance.csv; ablation/uncertainty plots

v

6) Similarity Analysis vs. Cultivars

• Normalize features (params and/or performance signatures)
• Distances: Euclid., Manhattan, Minkowski, Cosine, Mahalanobis
• Monotone similarity & stability check; Artifacts: similarity_matrix.csv

v

7) Validation & Policy Checks

• External/withheld validation; document fixed assumptions (e.g., root depth)
• Ethics & data: no new sampling; open code/data with DOI
• Artifacts: validation_report.md; DATA_AVAILABILITY.md

v

8) Reporting Package (PLOS ONE-ready)

• Methods: versions, seeds, designs, GA config, similarity rule
• Results: convergence, ideotype vs cultivars, sensitivity
• Supplement: bounds.yaml, best_theta.csv, morris_effects.csv, scripts

Reviewer’s flowchart interpretation after the manuscript’s integrated modeling framework that couples **CERES-Rice** mechanistic crop model with a **Genetic Algorithm (GA)** and a **Morris global sensitivity analysis** to propose a rice **ideotype** that jointly improves **harvest index** and **water-use efficiency (WUE)** across contrasting environments in Senegal. The workflow: define decision-variable bounds (CERES genetic parameters), run Morris to identify influential factors, optimize a composite objective (**HI-WUE**) with a GA, and then compare the optimized “virtual cultivar” to 21 field-characterized cultivars with multiple **similarity metrics** and reporting.

Major technical and reproducibility issues (publication-blocking under PLOS ONE criteria)

Reproducibility & model/software specification

Model/software specification not reproducible enough

- The manuscript does not state the **DSSAT/CERES-Rice version/build, operating system, compiler/runtime, or random seeds** used. This prevents exact reproduction (throughout Methods; "CERES-Rice" is invoked on p.2–3, 5–7 without version info).
- The paper relies on CERES-Rice (DSSAT), but I could not find the **exact model and executable versions** (e.g., DSSAT vX.Y.Z, CERES-Rice build/date), compile flags, or OS/environment details. Without this, results are not fully reproducible. The author should specify model versions, random seeds, platform, and full run scripts or a containerized environment.
- They state that each GA individual is defined over ranges "as shown in Fig. 4-b" but do not supply numeric bounds in text or a table; these must be tabulated for reproduction. The manuscript asserts reliance on "iterative calibration and validation," but procedural details (objective functions, calibration datasets, split strategy, stopping criteria) are not described.
- **Action:** Provide **exact versions** (e.g., DSSAT vX.Y, CERES-Rice build/date), OS, and **random seeds** for GA/Sobol/Morris, plus **scripts or a container** to reproduce runs.

Sensitivity analysis: insufficient specification

Morris method design parameters not fully specified

- You describe the algorithm (p.7), but the **numeric design choices** that determine accuracy/compute cost are missing: **grid level p, step size Δ, number of trajectories r** (p.7, l.32–37 mention the symbols but no values).
  The Morris design parameters (grid level *p*, step size Δ, number of trajectories *r*) and sampling design are not provided. Algorithm 1 is described conceptually, but numeric settings are missing; these determine both compute cost and inference quality and must be reported (with code producing the exact design).
- **Action:** Report **k, p, Δ, r**, sampling scheme, and provide the **exact design-generation script**.

Fitness definition ("HI-WUE") is ad hoc and needs justification and robustness

Fitness metric (HI-WUE) needs justification/robustness

- You define **HI-WUE = HI + 0.1×WUE** (Eq. (2), p.9, l.36). The weight **0.1** appears **arbitrary** (no scale normalization or trade-off analysis or multi-objective considerations). A sensitivity analysis to this weight (or a Pareto front presentation) is needed to show conclusions are robust.
- **Action:** (i) justify the weight by **normalizing** HI and WUE to comparable scales or (ii) present a **Pareto front** (HI vs WUE) and show that your chosen solution is robust to weights (e.g., 0.05–0.5).

Similarity/normalization method may invert interpretation

Similarity/normalization mapping is counter-intuitive

- The "average similarity" is computed as mean(1/(1+distance)) across metrics (Eq. 6). Then "Normalized Similarity = 1 – AvgSimilarity / max(AvgSimilarity)" (Eq. 7, page11, line 25). This mapping sets the **most similar items to 0** and **less similar to larger values**, which contradicts standard intuition and later narrative. At minimum, the mapping should be clarified or replaced with a monotone scaling such as (x−min)/(max−min).
- **Action:** Replace with a monotone scaling (e.g., **(x−min)/(max−min)**) so that **larger = more similar**; state clearly whether min/max are computed **per environment, per metric, or globally**.

Mixing heterogeneous distance metrics without standardization

Averaging heterogeneous distance metrics without standardization

- Averaging inverted distances from Euclidean, Manhattan, Minkowski(*p*=3), Cosine, and Mahalanobis assumes commensurate scales post-transform. This is debatable even with 1/(1+d). The author should (i) standardize each metric to [0,1] using min–max across the comparison set (or z-score before distance), (ii) provide a robustness check showing cultivar rankings are stable to metric choice, or (iii) justify a single principled metric (e.g., Mahalanobis).
- **Action:** Either (i) **standardize** each metric to [0,1] using the same cohort and show rankings are stable, or (ii) justify a single principled choice (e.g., **Mahalanobis** on z-scored features with a pooled covariance).

GA configuration vs. convergence evidence

- You state **20 generations, population 15, mutation probability 0.7** (p.8, l.35–36), but provide **no convergence traces, no seed control**, and **no sensitivity** to hyperparameters.
- **Action:** Provide **convergence diagnostics** (best/mean fitness per generation across **≥5 seeds**), and a brief sensitivity to pop size/generations/mutation.

Assumptions with strong influence not justified

- **Root depth fixed at 1 m** across environments is stated later in Results (p.23, l.33–37) but not motivated in Methods.
- **Action:** Either **justify** this with citations/calibration or include root-depth sensitivity.

Methods & algorithms (clarity/consistency)

- **Parameter symbol inconsistency (zero vs. letter O)**
  Genetic params use **P2O** elsewhere, but Methods define **P20** (zero) as critical photoperiod. Choose one (CERES uses **P2O**, letter O) and standardize.
- **GA parameter set size vs. stated factors**
  GA "individual" includes **8 parameters** {P1, P5, P2R, PHINT, P2O/0, G1, G2, G3}, but Morris section states **k = 11** input factors. Clarify which 3 are excluded from GA and why.

- **Typo in section header**
  "Ideotype through **AG** optimization" → **GA**.

- **Morris caption notation**
  Fig. 4 caption uses "**Rk=3**" and "**Rk=11**" (unclear). If you meant **r** (trajectories) and **k** (factors), rewrite (e.g., "r = 3, k = 11").

- **Normalization formula readability & behavior**
  The "Normalized Similarity = 1 − AvgSimilarity / max(AvgSimilarity)" line breaks mid-fraction and (as written) maps the **most similar to 0**. Consider a monotone scaling like (x−min)/(max−min)(x−\min)/(\max−\min)(x−min)/(max−min) and typeset on one line.

Results internal consistency

- **Root depth statement**
  "Root depth remained constant at **1 m** across regions" — given WUE focus, this fixed assumption should be signposted earlier in Methods and justified (or tested in sensitivity).

- **Unit + value coupling in percent summaries**
  "WUE increased by 6.58% (**5.83 Kg/mm**)" — mix of % change with absolute value plus wrong unit/capitalization. Use either percentage change only or give both change and unit-correct absolute value (kg ha^{-1} mm^{-1}).

Additional technical clarifications (strongly recommended)

- ### GA decision variables vs. Morris factors
  The GA optimizes **8** CERES parameters (e.g., P1, P2O, PHINT, P2R, G1, G2, G3, P5), while Morris states **k=11** factors (p.14). Please **list the exact GA variable set with numeric bounds** in a table and **explain which Morris-screened factors were omitted** from GA and why.

- ### Terminology/notation
  Use **P2O** (letter O) consistently; there is at least one **P20** (zero) on p.13, l.20.
  Section header typo: "**AG optimization**" → **GA optimization** (p.21, l.366; p.25, l.485).

- ### Units for WUE
  WUE is written multiple times as **kg/mm** or **Kg/mm** (e.g., p.16, l.40; p.22, l.14; p.23, l.9–11). Agronomic convention (yield per evapotranspiration) is **kg ha^{-1} mm^{-1}**. Please standardize and fix capitalization.

Additional important issues

- ### GA settings & convergence evidence:
  GA parameters are given (20 generations, pop. 15, mutation 0.7), but there is no convergence diagnostics, seed control, or sensitivity to these hyperparameters. Please request traces and

seeds; with a relatively small population, a 0.7 mutation rate risks non-convergence or noisy optima.

- ### Use of fixed root depth:
  Root depth is reported as **constant at 1 m across regions**, which can materially shape WUE/ET patterns. Either justify this constraint or analyze sensitivity to root depth.

- ### Data availability is good but could be strengthened for longevity:
  Data are on Mendeley Data and code on GitHub (good). For permanence and versioning, I recommend a **tagged release** archived with a DOI (e.g., Zenodo) and explicit instructions to reproduce the 5,692 runs.

- ### Ethics statement:
  Authors mark ethics as **N/A**. The work reuses previously published field data from Senegal (no new sampling). That is appropriate, but the Methods should explicitly say no new field collections were conducted and point to the prior study for permits/approvals.

- ### Preprint and prior posting:
  The manuscript cites a related **Research Square preprint (2025)** by the same group. PLOS ONE allows preprints, but please confirm overlap and ensure the present submission is the **Version of Record** with non-duplicative results; an explicit statement on overlap would help editorial assessment.

- ### English & style:
  The manuscript is generally readable but needs copyediting (e.g., inconsistent units "Kg/mm" vs "kg mm^{-1}"; "rainfeed" in captions should be "rainfed"). These are fixable at revision.

Positives

- Clear framing and dataset description (rainfed trials; use of SoilGrids and NASA POWER) with explicit references; this is a solid platform for the analysis.
- The data/code availability statement is present with live repository locations (see note above about archiving).
- The manuscript gives a transparent count of simulated cultivars and total runs, which is good practice.

English, style, and presentation (actionable edits with locations)

Language & style (precise locations)

- "a individual" → *an individual*. p. 14/26, l. 373.
- "which confirming" → *which confirms*. p. 14/26, l. 388.
- "number of grain" → *number of grains*. p. 14/26, l. 370.

- “a effective metric” → *an effective metric*. p. 17/26, l. 494.
- “The sensitivity analysis assess” → *assesses*. p. 12/26, l. 319.
- Truncated institution name (Acknowledgements)
  “School of Engineering at **Javer**…” appears cut off; likely **Javeriana**.

Figures, captions & labels

- Fig. 6 panel labels: “Biomass(Kg/H), Grain Yield(Kg/H), Number of Grains(#/H), Number of Tillers(#/H); panel (f) duplicated as *Anthesis* instead of *Maturity*. Use SI/area/time and fix (f) → *Maturity*. p. 12/26, l. 325–327 (caption line block). “Biomass(**Kg/H**)… Number of Grains(**#/H**)…” → use SI: **kg ha^{-1}**, **no. ha^{-1}**; fix capitalization and spacing.
- Fig. 5 caption: “rainfeed” → *rainfed*. p. 10/26, legend line.
- Fig. 9 caption: “rainfeed” → *rainfed*. p. 15/26, l. 399–401 (caption block begins at 399; caption text at 401).
- Section header “Ideotype through AG optimization” → …*GA*… (Genetic Algorithm). p. 14/26, l. 366.

Tables / variable lists

- **Variable alias duplication**
  In the variable list, “**Soil Sand (g/kg) – Soil Sand**” duplicates the name; provide the proper code alias (e.g., SLSA) or remove the repeat.
- **Mixed ASCII/Unicode exponents**
  Same table block mixes **cm3/cm3** with **cm^3/dm^3**. Standardize to Unicode with negative exponents (e.g., **cm^3 cm^{-3}**).

Units & symbols (standardize)

- WUE units shown as “kg/mm” (and “Kg/mm”). If WUE = (grain yield, kg ha^{-1}) / ET (mm), please write kg ha^{-1} mm^{-1} (lowercase “kg”). Occurs at p. 14/26, l. 383–384 and p. 15/26, l. 407–409.
- LAI units printed as “mm^2/mm^2” → use m^2 m^{-2} (dimensionless). p. 14/26, l. 387.
- Root density: “cm/cm^3” and “cm cm¯3” are mixed. Pick one (common in DSSAT papers: cm cm^{-3}). p. 14/26, l. 385–386; p. 15/26, l. 405–406.
- Hydraulic conductivity mixed as “cm h−1” vs “cm/h”. Use cm h^{-1} consistently. “cm/h” appears in Environments: p. 5/26, l. 100–102.
- Bulk density shown as “cg/cm^3” in the variables list; should be g cm^{-3}. p. 1/26, variable list lines.
- Space around “±”: write 0.50 ± 0.07 (not “0.50± 0.07”). p. 15/26, l. 401.

Notation & acronym hygiene

- Parameter P2O is mistyped as P20 in the GA individual definition. p. 8/26, l. 218. (Keep the letter “O”.)
- Throughout text the “AG”/“GA” inversion occurs in header; elsewhere “Genetic Algorithm (GA)” is correct. See header fix above.

Methods text (clarity/consistency)

- "Morris method … RSI defined …" block is fine; just ensure equation numbering and symbol spacing match journal style (thin spaces around fractions). p. 7/26, l. 188–193.
- GA settings are stated (gens = 20; pop = 15; mutation = 0.7). Good—just verify consistency with any plots reported. p. 8/26, l. 214–215.

References: formatting and completeness (PLOS ONE style)

- Journal title case: "PloS one" → *PLOS ONE*. Ref 7, p. 21/26.
- Journal name: "Plant Cell and Environment" → *Plant, Cell & Environment*. Ref 27, p. 23/26.
- Author spelling: "Saxtonn K." → *Saxton* (W. J. Rawls, D. L. Brakensiek, K. Saxton). Ref 37, p. 23/26.
- Avoid ALL CAPS (e.g., "JOURNAL OF EXPERIMENTAL BOTANY") in reference list.
- Ensure diacritics render (e.g., Martínez, Benítez-Restrepo) throughout references.

Punctuation

"**et al..**" (double period) in at least two entries (p.30, l.32; p.31, l.3) → "**et al.**".

Completeness

- Data-source references (e.g., **NASA POWER**) entry is incomplete (currently read "Data; 2017."). Provide the **full citation/URL/DOI** Add authors/maintainer, title, access date/URL or DOI per PLOS style. Ref 38, p. 23/26.
- Check any chapter/edited volume citations include **editors, publisher, city, pages**, and DOI/ISBN.

Incomplete items (missing venue/DOI/publisher)

- **Ref. 19** "Testing of crop Models … 2014;." → missing journal/book/proceedings and DOI.
- **Ref. 18** "Cereal growth, development and yield. Understanding options for agricultural production. 1998; p. 79–98." → add editors, publisher, city, and ISBN/DOI if available.
- **Ref. 38,** p. 23/26. "NASA POWER Data; 2017" data-source reference entry is incomplete (currently read "Data; 2017."). Provide the **full citation/URL/DOI**. Add authors / maintainer, title, access date/URL or DOI per PLOS style. Ref 38,
- **Accents & name consistency**
  In the text/acknowledgements "Gerardeaux" vs References "Gérardeaux" — pick one (with or without diacritics) and apply consistently across text and references.
- **Data/code availability vs references**
  You correctly cite Mendeley Data and GitHub in both "Data and Code Availability" and pre-References boilerplate; avoid duplication or ensure the wording is identical.
- Cross-check that **every reference is cited in text** and vice versa.

Preprint policy

- You cite a **2025 Research Square preprint** by (presumably) the same group (Ref 39, p.24, l.5–9). PLOS ONE allows preprints; please **state the overlap** and ensure this submission is the **version of record** with non-duplicative results.

Algorithm/math clarity

- **Normalized similarity definition**
  As written: "similarity = 1 − avg distance / max(avg distance)" (line break mid-formula). Consider typesetting clearly and stating the normalization set (per-v, per-metric, or global).

Small style polish (optional but helpful)

- Use narrow non-breaking space around % (journal-dependent), or just a space: "23.9%" is fine; be consistent. Example cluster: Fig. 8 paragraph.
- Use a consistent en-dash (–) for numeric ranges (e.g., "670–780 nm") and minus signs in exponents (m^{-2}). You already do this in several places; just normalize.

Data & code availability (meets policy, can be strengthened)

- You provide **Mendeley Data** and **GitHub** links (p.6 and p.21 line 622). For long-term reproducibility, please make a **tagged release archived with a DOI (e.g., Zenodo)** and point the Data Availability statement to that DOI along with Mendeley.

Ethics and research integrity

- Methods indicate **no new field data collection** (re-use of Senegal trials). Please make explicit in Methods that **no new sampling** occurred in this study and cite the original study(ies) for permits/approvals, aligning with PLOS ONE's ethics criteria.

Recommendation and revision checklist

Recommendation: Major Revision (publishable in principle once technical soundness and reproducibility are fully addressed).

Minimum revisions for PLOS ONE acceptance:

1. Specify **DSSAT/CERES-Rice versions**, OS, seeds; provide **scripts or a container**.
2. Report numeric **Morris design** parameters (**k, p, Δ, r**) and attach the design-generation code.
3. Justify **HI-WUE** weight (**0.1**) or replace with a **Pareto analysis**; add a robustness check.
4. Fix the **similarity normalization** so **higher = more similar**; clarify normalization scope; show stability across metrics.
5. Either **standardize** heterogeneous distance metrics before averaging or justify a single metric (with a robustness check).

6. Provide **GA convergence diagnostics** and **seeded replicates**; briefly assess sensitivity to GA hyperparameters.
7. Justify (or analyze sensitivity to) **fixed root depth**; move the assumption into Methods.
8. Implement the **English/style** fixes enumerated above (units, labels, spelling, spacing, header typos).
9. Bring the **References** into PLOS style (journal names, author spellings/diacritics, punctuation, completeness) and ensure 1-to-1 mapping with in-text citations.
10. Strengthen **Data Availability** with a **versioned DOI** for code.

I do hope this detailed feedback helps you bring the manuscript into full alignment with PLOS ONE's standards for technical rigor, clarity, and reproducibility. Please, find here a detailed check list to help you with the corrections.

With best regards,

PLOS One Reviewer

AUTHOR REVISION CHECKLIST — PLOS ONE (Modeling Study)

Use this checklist to ensure your manuscript satisfies PLOS ONE's acceptance criteria. Check off each item and insert page/line references and links to artifacts where indicated.

1. Manuscript metadata (fill in)

Title: ____

____

Manuscript ID: ______________________________ Version: ___________________________

Scientific article pagination starts at page 1/26 (PDF page 8). Cite right-margin line numbers in responses.

2. Reproducibility & Transparency

- ☐ Model/software versions are specified (DSSAT/CERES build/date, OS, compiler/runtime, Python/R packages).
- ☐ Random seeds for Morris and GA are declared and applied.
- ☐ Executable/run harness provided (scripts or container) to reproduce all runs.

Artifacts/links: ENVIRONMENT.md, env.yaml or Docker/Singularity, run scripts, version tags/DOIs.

3. Methods Completeness

- ☐ Morris design fully reported: number of factors k, grid levels p, step Δ, trajectories r; sampling scheme described.
- ☐ CSV of the exact Morris design and computed μ* and σ are provided.
- ☐ GA decision variables are listed with NUMERIC bounds and agronomic constraints (e.g., P1, P2O, P2R, P5, PHINT, G1–G3).
- ☐ Objective function defined and justified: scaling of HI/WUE; weight α justified OR Pareto (NSGA-II) provided.
- ☐ GA configuration reported: population, generations, crossover, mutation, elitism; ≥5 seeded runs; convergence traces included.
- ☐ High-leverage assumptions (e.g., root depth) justified or sensitivity added; moved to Methods.
- ☐ Similarity analysis uses monotone normalization (larger = more similar) and states normalization scope (per-env vs global).
- ☐ If averaging heterogeneous distances, metric standardization (z-score or [0,1]) and ranking stability are shown, OR a single principled metric is justified.

Artifacts/links: morris_design.csv, morris_effects.csv, bounds.yaml, objective.yaml, convergence plots, similarity_matrix.csv.

4. Results & Uncertainty

- ☐ Uncertainty (CIs/SDs) reported for HI, WUE, yield across sites/years and across GA seeds.
- ☐ Local sensitivity around the ideotype (±10% parameter ablations) demonstrates robustness.

5. Figures, Tables, Equations

- ☐ Units standardized: WUE (kg ha^{-1} mm^{-1}), Yield/Biomass (kg ha^{-1}), Counts (no. ha^{-1}), LAI (m^2 m^{-2} or –), Ksat (cm h^{-1}), BD (g cm^{-3}).
- ☐ Parameter codes and headings consistent (P2O not P20; GA acronym in headers).
- ☐ Figure legends/captions corrected (e.g., Fig. 6f = Maturity; "rainfed" spelling; spaces around ±; axes with units).
- ☐ Equations numbered; symbols defined at first use; similarity equation typeset on one line.

6. English, Grammar, Style

- ☐ Article/verb agreement and wording corrected (e.g., "an individual point", "analysis assesses").
- ☐ Terminology consistent (ideotype, Genetic Algorithm (GA), CERES-Rice).
- ☐ Subjective adjectives replaced by metrics (e.g., provide RMSE/CI rather than "excellent").

Artifacts/links: tracked changes or change-log mapping edits to page/line numbers.

7. References (PLOS style & completeness)

- ☐ Journal titles/capitalization corrected (PLOS ONE; Plant, Cell & Environment; Journal of Experimental Botany).
- ☐ Author spellings/diacritics fixed (e.g., Saxton K.E.; Martínez).
- ☐ No punctuation errors (e.g., "et al." not "et al..").
- ☐ Data-source citations complete with URL/DOI and access dates (e.g., NASA POWER).
- ☐ One-to-one mapping between in-text citations and reference list (no uncited or missing references).

8. Ethics & Data Availability

- ☐ Ethics statement clarifies no new field sampling; prior permits/approvals cited if applicable.
- ☐ Code/data publicly available with a versioned DOI (Zenodo/OSF); Data Availability statement updated.

Contents

Table of Contents

Dear Author, ... 1

Summary of the study (what the paper does) 1

Manuscript metadata 1

Major technical and reproducibility issues (publication-blocking under PLOS ONE criteria) 3

Reproducibility & model/software specification 3

Sensitivity analysis: insufficient specification 3

Fitness definition ("HI-WUE") is ad hoc and needs justification and robustness 3

Similarity/normalization method may invert interpretation 4

Mixing heterogeneous distance metrics without standardization 4

GA configuration vs. convergence evidence 4

Assumptions with strong influence not justified 4

Methods & algorithms (clarity/consistency) 4

Results internal consistency 5

Additional technical clarifications (strongly recommended) 5

- GA decision variables vs. Morris factors 5
- Terminology/notation 5
- Units for WUE 5

Additional important issues 5

- GA settings & convergence evidence: 5
- Use of fixed root depth: 6
- Data availability is good but could be strengthened for longevity: 6
- Ethics statement: 6
- Preprint and prior posting: 6
- English & style: 6

Positives 6

English, style, and presentation (actionable edits with locations) 6

Language & style (precise locations) 6

Figures, captions & labels 7

Tables / variable lists 7

Units & symbols (standardize) 7

Notation & acronym hygiene 7

Methods text (clarity/consistency) 8

References: formatting and completeness (PLOS ONE style) 8

Algorithm/math clarity 9
Small style polish (optional but helpful) 9
Data & code availability (meets policy, can be strengthened) 9
Ethics and research integrity 9
Recommendation and revision checklist 9
AUTHOR REVISION CHECKLIST — PLOS ONE (Modeling Study) 11
1. Manuscript metadata (fill in) 11
2. Reproducibility & Transparency 11
3. Methods Completeness 11
4. Results & Uncertainty 11
5. Figures, Tables, Equations 12
6. English, Grammar, Style 12
7. References (PLOS style & completeness) 12
8. Ethics & Data Availability 12
Contents 13
