# Supplementary material for: Decoding living systems: Reassessing crop model frontiers via biological dynamics and optimized phenotype
Source: PLoS One. 2026 Mar 11;21(3):e0343530. doi: 10.1371/journal.pone.0343530 (PMC12978445; doi:10.1371/journal.pone.0343530)
Supplement: Attachment — Submitted filename: 1.Response to Reviewers 29_12_2025.pdf [file pone.0343530.s008.pdf]

PEER REVIEW _ PLOS ONE (08/09/2025)

1. Is the manuscript technically sound, and do the data support the conclusions? The manuscript must describe a technically sound piece of scientific research with data that supports the conclusions. Experiments must have been conducted rigorously, with appropriate controls, replication, and sample sizes. The conclusions must be drawn appropriately based on the data presented.
Reviewer #1: Yes
Reviewer #2: Yes
Reviewer #3: Partly

2. Has the statistical analysis been performed appropriately and rigorously?
Reviewer #1: Yes
Reviewer #2: Yes
Reviewer #3: No

3. Have the authors made all data underlying the findings in their manuscript fully available? The PLOS Data policy requires authors to make all data underlying the findings described in their manuscript fully available without restriction, with rare exception (please refer to the Data Availability Statement in the manuscript PDF file). The data should be provided as part of the manuscript or its supporting information or deposited to a public repository. For example, in addition to summary statistics, the data points behind means, medians and variance measures should be available. If there are restrictions on publicly sharing data—e.g. participant privacy or use of data from a third party—those must be specified.
Reviewer #1: No
Reviewer #2: Yes
Reviewer #3: Yes

4. Is the manuscript presented in an intelligible fashion and written in standard English? PLOS ONE does not copyedit accepted manuscripts, so the language in submitted articles must be clear, correct, and unambiguous. Any typographical or grammatical errors should be corrected at revision, so please note any specific errors here.
Reviewer #1: Yes
Reviewer #2: Yes
Reviewer #3: No

PLOS authors have the option to publish the peer review history of their article (what does this mean?). If published, this will include your full peer review and any attached files. If you choose "no", your identity will remain anonymous, but your review may still be made public. ***Do you want your identity to be public for this peer review?*** *For information about this choice, including consent withdrawal, please see our Privacy Policy.*
Reviewer #1: **Yes:** Dr. Shahzad Akhtar
Reviewer #2: No
Reviewer #3: **Yes:** Ronald Maldonado Rodriguez

AUTHORS' RESPONSE TO PEER REVIEW COMMENTS

I sincerely thank the reviewers for their thorough evaluation and constructive feedback. Their insightful comments have significantly improved the clarity, rigor, and presentation of this manuscript. I have carefully addressed each point raised, incorporating substantial revisions to the methodology description, statistical analysis, and figure quality. Below, I provide detailed responses to each comment, with all modifications highlighted throughout the revised manuscript.

REVIEWER #1

Review Report Title: Mechanistic crop modelling and AI for ideotype optimization: Crop-scale advances to enhance yield and water use efficiency

The manuscript presents a novel integration of mechanistic crop modeling (CERES-Rice) and AI(Genetic Algorithm) for ideotype optimization. It is methodologically sound, well-organized, and offers valuable insights for precision agriculture. Major Comments with Line References

Final Recommendation: Major Revision

The study is innovative and promising but requires additional methodological justification, statistical clarity, and formatting refinements before acceptance.

**Comment**	**Answer**
**1.1** Line 51–56: Justify the selection of 1,884 virtual cultivars and 5,692 runs; clarify stopping criteria and convergence.	The computational exploration was expanded to 5,364 virtual cultivars across 40 generations, systematically covering four distinct environments representing 89% of the regional cultivation area. Convergence analysis (Supplementary Figure S1) demonstrates fitness stabilization by generations 10-23 across environments, validating adequate parameter space exploration.<br><br>Changes in manuscript:<br>- Abstract (Lines 12-15): Updated to "5,364 virtual cultivars across 40 generations"<br>- Methods (Lines 60-63): Specified systematic environment selection and coverage<br>- Table S3: Complete optimal genetic coefficients with convergence generation<br>- Figure S1: Convergence trajectories showing stabilization for all four environments
**1.2** Lines 208–215: Genetic Algorithm parameters (population size = 15, generations = 20) seem low; justify or consider alternatives.	GA parameters were revised to 40 generations (increased from original 20), with population size = 15 and mutation rate = 0.7. Convergence

	analysis (Figure S1) shows fitness trajectories stabilized by generations 10-23 across environments, with stable plateaus through generation 40. The high mutation rate facilitated broad search space coverage.<br>Changes in manuscript:<br>- Abstract (Line 13): "40 generations"<br>- Table S3: Convergence generation for each environment (10-23)<br>- Figure S1: Complete convergence plots demonstrating adequate exploration
**1.3** Algorithm 2: No mention of overfitting control; suggest cross-validation or bootstrapping.	Cross-validation and bootstrapping are designed for empirical models fitted to data. CERES-Rice is a mechanistic model encoding crop physiology through differential equations. Key distinctions:<br><br>(i) Optimized parameters are genetic coefficients with physiological meaning (thermal time requirements, grain characteristics, tillering), not empirical regression weights.<br>(ii) Each genotype is evaluated across 12 environment-year combinations (4 environments × 3 years), providing inherent temporal and spatial validation.<br>(iii) The fitness function (HI + WUE_normalized) rewards physiologically consistent multi-year performance.<br><br>Robustness is demonstrated through:<br>- Stable convergence patterns (Figure S1)<br>- Physiologically plausible parameter values (Table S3)<br>- Similarity to real cultivars (70.7% maximum similarity)<br><br>No changes required—mechanistic vs. empirical distinction clarified in Discussion (Lines 589-602).
**1.4** Line 52 & 417: Number of environments (n=4) may limit generalizability; validate across more diverse settings.	The four environments were systematically selected through GMM-based environmental classification (Correa et al., 2025), collectively covering 89% of the Casamance and Eastern Senegal cultivation area:

	- Environment 1 (30%): High water retention, high precipitation<br>- Environment 2 (18%): Medium retention, low precipitation<br>- Environment 3 (21%): Medium retention, high precipitation<br>- Environment 4 (20%): Low retention, low precipitation<br><br>These environments capture critical gradients in soil water-holding capacity and precipitation regimes.<br><br>Changes in manuscript:<br>- Abstract (Lines 15-16): "covering 89% of the cultivation area"<br>- Methods (Lines 144-169): Complete GMM classification description<br>- Figure 3: Environmental categories visualization<br>- Table S3: Environment descriptions with soil-climate characteristics
**1.5** Fig. 6 & Lines 327–366: Add confidence intervals or significance testing to support sensitivity rankings.	The reviewer's suggestion is appreciated. Sensitivity analysis was performed using 20 independent replications with randomized parameter sequences, providing robust confidence intervals for parameter rankings. The narrow confidence intervals (mean 95% CI width = 0.04) confirm that sensitivity rankings are robust features of the model structure rather than random fluctuations.<br><br>Changes in manuscript:<br>- Abstract (Line 11): Added "robust rankings (95% CI width = 0.04)"<br>- Results section: Added "mean 95% CI width = 0.04" with interpretation<br>- Table S1: Complete RSI statistics (mean ± SD) for all 11 parameters × 6 outputs<br>- Figure 6 caption: Added "Values represent means across 20 independent replications; complete statistics with 95% CI in Table S1"<br>- Supplementary Material: Methodology description detailing the 20 replications approach

**1.6** Fig. 7 & 8: Report statistical significance of correlation results.	This suggestion is appreciated. Statistical significance has been added to all correlation results.<br>Key findings with significance levels:<br>- HI-WUE vs grain yield: $R^2 = 0.78–0.86$, $p < 0.001$<br>- HI-WUE vs harvest index: $R^2 = 0.88–0.97$, $p < 0.001$<br>- HI-WUE vs water use efficiency: $R^2 = 0.86–0.93$, $p < 0.001$<br>- Biomass, LAI, root density: $R^2 < 0.08$ (practically negligible despite statistical significance due to large sample size, n = 5,364)<br><br>Changes in manuscript:<br>- Figure 7 caption: Added "($R^2 = 0.78–0.86$, $p < 0.001$)" for key correlations<br>- Results section: p-values added to all correlation statistics<br>- Table S2: Complete correlation matrix with significance levels (*** $p < 0.001$, ** $p < 0.01$, * $p < 0.05$, ns $p \geq 0.05$)<br>- Table S2 caption: Note added explaining that correlations with $R^2 < 0.10$ are practically negligible despite statistical significance due to large sample size
**1.7** Line 423–426: Promising cultivar claims based on similarity metrics alone; field validation needed.	The 21 field-characterized cultivars serve as validation reference—their genetic coefficients were derived from 3 years of field experimentation (2012-2014) as reported in Gerardeaux et al. (2021). Similarity analysis identifies which existing, field-validated cultivars most closely match computational optima, providing breeding programs with actionable starting points.<br><br>The framework quantifies:<br>- Global similarity: WAB56-50 (70.7%), DKAP2 (67.2%)<br>- Genetic gap: 22-30% between current germplasm and computational optima<br>- Parameter-specific distances enabling targeted

	breeding interventions<br><br>Full field validation of optimized ideotypes would require multi-location trials beyond the scope of this computational study, but the identified cultivars (WAB56-50, DKAP2) represent validated genetic backgrounds closest to theoretical optima.<br><br>Changes in manuscript:<br>- Results (Lines XX): Similarity analysis with specific percentages<br>- Discussion (Lines XX): Breeding roadmap interpretation<br>- Figures E9, E9b, E10: Visual representation of similarity and genetic gaps
**1.8** Line 457: Present PCA variance (e.g., 86.05%) in tabular form.	PCA variance (85.8%) is reported in Results text and Figure E9 caption. Component breakdown:<br>- PC1: 57.2%<br>- PC2: 16.8%<br>- PC3: 11.9%<br>- Total: 85.8%<br><br>Changes in manuscript:<br>- Results (Line XX): "PCA conducted on these genetic parameters accounts for 85.8% of total variance"<br>- Figure E9 caption: "(85.8% variance explained)"<br><br>If tabular format is preferred, this can be added to Supplementary Material as Table S4.
**1.9** Lines 1–60 (Abstract): Too dense; trim to emphasize key findings and novelty.	This feedback is appreciated. The abstract has been restructured to improve clarity and emphasize key contributions. The revised abstract follows a clearer progression: (1) Context and gap: Process-based models address AI black-box limitations (2) Framework contribution: Three integrated analytical layers (3) Key findings: Sensitivity analysis, optimization results, similarity analysis (4) Impact: Compression of breeding discovery phase and extensibility to other biological scales Changes in manuscript: - Abstract reorganized into distinct conceptual

	blocks with clearer topic sentences - Redundant technical details moved to Methods section - Key quantitative findings retained (5,364 cultivars, 40 generations, 70.7% similarity, 22–30% genetic gap) - Novelty statement strengthened in opening and closing sentences
**1.10** Lines 545–560 (Conclusion): Avoid repetition; stress broader implications for breeding.	This observation is appreciated. The Conclusion has been restructured to eliminate redundancy and emphasize broader implications. The revised version includes three distinct subsections: (1) "AI-driven optimization of biological processes" — methodological contribution (2) "Case study findings" — three actionable insights without repeating Results (3) "Modeling living systems: limitations and horizons" — broader implications for breeding and extension to cellular scales<br><br>***Changes in manuscript***: - Conclusion reorganized into three focused subsections - Repetition of quantitative results already presented in Results section removed - Explicit breeding roadmap added: "transforming vague improvement goals into measurable breeding targets" - Discussion of extensibility to cellular systems and future biological applications expanded - Critical reflection on model boundaries and the gap between biological and computational expertise added
**1.11** Line 376–393: Break long paragraphs for better readability.	This suggestion for improving readability is appreciated. Long paragraphs have been divided into focused subsections with clear topic sentences.<br>***Changes in manuscript***:<br>- Results section reorganized with subheadings: "Biomass and yield sensitivity", "Phenological timing", "Thermal stress parameters"<br>- Discussion section restructured with subheadings: "Sensitivity analysis as biological validation", "From parameter influence to phenotypic optimization", "Bridging computational ideotypes to breeding targets", "Model boundaries and future directions"<br>- Long paragraphs divided to maintain one main

	idea per paragraph<br>- Transitional sentences added to maintain logical flow between sections
**1.12** Lines 20–40: Include foundational references (e.g., Donald's ideotype concept).	Donald's foundational work on ideotype breeding has been included in the Introduction, contextualizing the historical evolution from empirical breeding to deliberate ideotype design.<br><br>Changes in manuscript:<br>- Introduction (Lines 34-44): "Traditional breeding relies on empirical selection: correcting defects or identifying high-yielding variants through 10–15 years of iterative field trials. This approach selects the best available genotypes but does not define optimal phenotypes—breeders know what they find, not what they seek (Donald, 1968). C. M. Donald proposed a third approach: systematic ideotype design grounded in biological process-based models..."<br>- References: Donald, C.M. (1968). The breeding of crop ideotypes. Euphytica, 17(3), 385-403.
**1.13** Line 68–87: Avoid excessive reuse of [32]; clarify citation purpose.	This issue is acknowledged. The citation pattern has been revised to provide clearer attribution and reduce over-reliance on a single reference.<br><br>Changes in manuscript:<br>- Citations diversified throughout the Introduction, adding foundational references for each concept:<br>- Donald (1968) for ideotype breeding concept<br>- Tardieu et al. (2017), Hammer et al. (2019) for genotype-environment modeling<br>- Additional references for AI applications in agriculture<br>- Citation [32] (Correa et al., 2025) now cited specifically for GMM-based environmental classification methodology<br>- Each citation serves a distinct purpose: methodological precedent, conceptual foundation, or data source
**1.14** Line ~500: Ensure ethics statement is clearly included in manuscript.<br>**1.15** Line 536–541: Briefly discuss potential	An Ethics Statement section has been added addressing both current study and future considerations:

future ethical considerations.	Current study: Uses publicly available datasets and computational simulations. No human subjects, animals, or protected species involved. Field data collected under established agronomic protocols.<br><br>Future applications: Framework extension to cellular/biomedical systems would require appropriate ethical oversight, IRB compliance, and bioethics guidelines.
**General Notes**<br>**1.16** Ensure all figures (e.g., 7 & 8) are fully labeled with units.	This attention to detail is appreciated. All figures have been reviewed and updated to ensure complete labeling with appropriate units.<br><br>Changes in manuscript:<br>- Figure 6: Units added to all axes (RSI: dimensionless, 0–1 scale)<br>- Figure 7: Units added to axes (HI-WUE: dimensionless; Grain yield: kg/ha; Biomass: kg/ha; WUE: kg/mm)<br>- Figure 8: Units added (Yield: kg/ha; HI: dimensionless; WUE: kg/mm; Cycle: days; GDD values for genetic coefficients)<br>- All figure captions reviewed for consistency in unit reporting
**1.17** Include summary statistics (e.g., mean ± SD) in figure captions.	This suggestion for improving data transparency is appreciated. Summary statistics have been added to relevant figure captions.<br><br>Changes in manuscript:<br>- Figure 6 caption: Added "Values represent means across 20 independent replications; complete statistics with 95% CI in Table S1"<br>- Figure 7 caption: Added correlation statistics with significance levels "(R^2 = 0.78–0.86, $p < 0.001$)" and reference to Table S2<br>- Figure 8 caption: Added yield ranges and efficiency metrics for each environment<br>- Figure 9 caption: Added "85.8% variance explained" and similarity percentages for top cultivars<br>- All summary statistics referenced to

	supplementary tables for complete data access
**1.18** Add version numbers of DSSAT, Python packages, etc.	Software version numbers are specified in the manuscript:<br>- DSSAT v4.8 (Lines 173)<br>- MATLAB R2024b (Line 174-175)<br>- R v4.4.2 (Line 175)<br>- Windows 11 Professional Build 26100.7171 (Lines 171-173)<br>- Intel Core i7-12700H processor, 32 GB RAM (Lines 171-172)
**1.19** Include a reproducibility checklist or flowchart as supplementary material.	This suggestion to enhance reproducibility is appreciated. The PLOS ONE Modeling Study Checklist has been addressed comprehensively:<br>**Reproducibility & Transparency**<br>• Software versions specified: DSSAT v4.8, MATLAB R2024b, R v4.4.2, Windows 11 Professional Build 26100.7171, Intel Core i7-12700H processor, 32 GB RAM<br>• Code repository: https://github.com/EdgarStevenC/Crop-Growth-Modelling (CC BY 4.0)<br>• Data repository: Mendeley Data DOI: 10.17632/msg7bfg5t6.1<br>**Methods Completeness**<br>• Morris design: k = 11 factors, step sizes (Δ) defined in Figure 4b, r = 20 trajectories<br>• GA configuration: population = 15, generations = 40, mutation rate = 0.7, 8 decision variables (P1, P5, P2R, PHINT, P2O, G1, G2, G3) with numeric bounds in Figure 4b<br>• Objective function: HI-WUE composite metric defined and justified<br>• Convergence traces: Figure S1 showing stabilization across all environments<br>**Results & Uncertainty**<br>• Sensitivity analysis: 20 replications with 95% CI (mean width = 0.04), Table S1<br>• Correlation statistics: R^2 with p-values, Table S2<br>• Optimization: Convergence generation

	reported for each environment (10–23), Table S3<br>**Figures, Tables, Equations**<br>• Units standardized throughout (kg/ha, kg/mm, GDD, days)<br>• All equations numbered and symbols defined<br>• Figure captions include summary statistics<br>**Ethics & Data Availability**<br>• Ethics statement included<br>• All code and data publicly available with DOI<br>**Changes in manuscript:**<br>• Supplementary Material expanded with Tables S1–S3 and Figure S1<br>• Methods section details Morris and GA parameters<br>• Data and Code Availability section includes versioned DOI

REVIEWER #2

Dear author, On the whole, I enjoyed reading your paper, and the detailed modelling and statistical approach taken. I do however feel beyond the results there is not enough insight (yet) in the discussion, and would like you to expand that a little bit, and make the paper less descriptive. See my comments below.

Comment	Answer
**2.1 Methods:** So regarding the AI model, basically the starting population is 166? (Delta 1 to 6 and 11 parameters? But how does the AI know the WUE and HI? Is the input data include the model output values of WUE and HI that correspond to each of the Fig 4B combinations? Maybe clarify the above a little bit please.<br>Line 247: the fitness factor here is HI-WUE? Why are 5 methods of dissimilarity testing are needed??? You also mention “virtual cultivars”. I thought only on virtual cultivar, the best from the Genetic Algorithm output, is compared to the 21 “real” cultivars? I feel some extra clarification required here too.	This clarification request is appreciated. The methodology has been expanded to address each point:<br>**Genetic Algorithm structure:** The GA operates with a population of 15 individuals per generation across 40 generations—separate from the Morris sensitivity analysis (Figure 4b), which defines parameter bounds for the 11 genetic coefficients.<br>**HI and WUE computation:** These metrics are not inputs to the GA. Each candidate solution (a set of 8 genetic coefficients) is evaluated by executing a complete CERES-Rice simulation. HI and WUE are calculated from simulation outputs:<br>• HI = Grain Yield / Total Aboveground Biomass

<table>
<tr><td></td><td>

- WUE = Grain Yield / Total Evapotranspiration

The fitness function is therefore computed after each simulation, not provided as input data.

Fitness function rationale: The composite HI-WUE index integrates harvest index and water use efficiency (both normalized to [0,1]), rewarding genotypes that efficiently convert biomass to grain while minimizing water consumption. This dual-objective approach avoids the limitation of optimizing WUE alone, which can favor low-yielding drought-stressed plants.

Multiple distance metrics: Three complementary metrics ensure robust cultivar ranking:

- Euclidean: geometric distance in parameter space
- Manhattan: coordinate-wise absolute differences
- Cosine: angular similarity between coefficient vectors

Consensus across metrics confirms that breeding recommendations are robust to methodological choice.

Virtual cultivars versus ideotypes: The GA explored 5,364 virtual cultivars across 40 generations, yielding four optimized ideotypes (one per environment). All four ideotypes (ID1–ID4) are compared against 21 field-validated cultivars—not a single ideotype.

Changes in manuscript:

- Methods: Explicit explanation of the simulation-evaluation loop added
- Algorithm 2: Clarified that fitness evaluation requires CERES-Rice execution
- Methods: Justification for multiple distance metrics added
- Terminology: Consistent distinction between "ideotype" (optimized solutions) and "virtual cultivar" (all GA-evaluated candidates)

</td></tr>
<tr><td>2.2 Results: Fig 6 RSI axis label sometimes over</td><td>This feedback for improving readability is</td></tr>
</table>

imposed on axis label Maybe possible to make Results shorter and incorporate some conclusion statements after each section? Like at tend of results what characterizes the sum of the changes required in the cultivars? Later flowering and panicle initiation etc….in simpler words.	appreciated.<br>Figure 6: Axis labels have been repositioned to avoid overlap with tick marks.<br>*Section conclusions:* Summary statements have been added at the end of each Results subsection, synthesizing findings in accessible terms:<br><br>*After Sensitivity Analysis:* "Phenological parameters (P1, PHINT) control developmental timing, while reproductive parameters (G3, G2) govern yield components—providing the mechanistic basis for targeted optimization."<br>*After Optimization:* "Two adaptive strategies emerged: extended growth cycles for favorable environments and shortened drought-escape phenology for water-limited conditions."<br>After Similarity Analysis: "WAB56-50 and DKAP2 emerge as priority breeding candidates, with phenological parameters requiring major adjustment while reproductive traits are already near-optimal."
**2.3 Discussion:** Line 499: Why would lower humidity be better for photosynthesis? Line 511: Why did the ideotype be better at conserving water, do you think? So far Discussion is just rehashing results.<br>Line 534-535: (SST)<br>When you signing critical for aligning crop development with environmental conditions, what do you mean exactly?<br>How does the alignment help? Please expand on this, explain then mechanisms. **There is very little physiology in the discussion so far. Line**<br><br>548: Phenological parameters being influential is not a groundbreaking finding, but you need to incorporate this **knowledge with the environment** and use it to **explain physiological** responses that are specific to environment or genotype. I also think you need to discuss, a bit, WUE itself and how it **can be counterproductive**, and how maybe incorporating with HI can be beneficial.	These observations highlighting the need for deeper physiological grounding are appreciated. The Discussion has been substantially expanded to address each point:<br>*Humidity and photosynthesis*:<br>The original statement required clarification. The relationship operates through vapor pressure deficit (VPD)—the driving force for transpiration. Northern environments exhibit VPD of 1.19 kPa versus 0.67 kPa in the south, representing 77% greater atmospheric water demand (Lines 475–481; Supplementary Note: VPD Calculation). This elevated VPD accelerates soil water depletion during the growing season, explaining why water limitation—not humidity per se—constrains productivity in northern environments.<br><br>*Water conservation mechanisms*:<br>The optimized ideotypes achieve higher WUE through two complementary mechanisms:<br>Phenological escape: Shortened cycles (100–103 days) complete grain filling before late-season

	drought intensifies<br>Improved partitioning: Higher HI (0.55–0.58) reduces transpirational cost of non-reproductive biomass<br>Phenological alignment:<br>"Alignment" refers to synchronizing crop water demand with water availability. The optimization positioned flowering—the most drought-sensitive stage—during periods of adequate soil moisture. In water-limited environments, this required compressing vegetative duration (reduced P1) and accelerating grain filling (reduced P5), completing reproduction before soil water exhaustion.<br>*WUE limitations and HI-WUE integration:*<br>WUE alone can mislead breeding programs: drought-stressed plants with minimal growth may achieve high WUE while producing negligible yield. The HI-WUE composite metric excludes these "false positives" by rewarding only genotypes combining efficient water use with effective grain formation.<br><br>*Changes in manuscript:*<br>Discussion (Lines 475–481): VPD-based mechanistic explanation added with quantitative values (1.19 vs 0.67 kPa)<br>Supplementary Material: Added "Supplementary Note: VPD Calculation" with Tetens equation and climate-specific calculations<br>Discussion: Mechanistic interpretation of drought-escape strategy expanded<br>Discussion: Explicit definition of "phenological alignment" with physiological basis<br>Discussion: WUE limitations and HI-WUE integration rationale added<br>References: Added Tetens (1930) and Monteith & Unsworth (2008)
**2.4** Finally, ***I understand the focus of this paper is methodological*** but as shown by my comments above you need a more physiologically grounded story in your conclusion and discussion so ***we know how your modelling approach***	This constructive feedback is deeply appreciated and addresses a central challenge in process-based modeling research.<br>The reviewer correctly identifies that the paper's focus is methodological. However, the

contributed to our understanding *of what's required for growth in theregion of your study*. For example, what other traits, other than the ones you tested, **do you think can be influential to improve crops in your region?** Use your modelling and the significant parameters to ruminate on that and incorporate a more physiological understanding specific to your environment.	contribution extends beyond methodology to a critical examination of how process-based models are employed in agricultural research—a reflection presented in the Discussion section "Modeling living systems: limitations and horizons" (Lines 565–580).<br>This section addresses precisely the concern raised: the gap between model application and biological understanding. As stated in the manuscript: "Platforms like DSSAT—integrating models such as CERES-Rice—have served as frameworks for decades, enabling agronomic research worldwide. Yet widespread adoption has paradoxically constrained scientific progress: users apply these models as established tools rather than testable hypotheses, accepting default parameterizations without questioning underlying assumptions. Biological expertise without computational scrutiny accepts model outputs uncritically; engineering expertise without biological insight optimizes parameters blindly."<br>This interdisciplinary framework—integrating sensitivity analysis, optimization, and similarity analysis—was designed to bridge this gap. The physiological insights emerge not from describing model outputs, but from interrogating model structure:<br>**Physiological contributions specific to the study region:**<br>(1) *Phenological alignment as drought adaptation*: The optimization revealed that successful adaptation in Casamance and Eastern Senegal requires phenological plasticity—the capacity to modulate developmental timing according to water availability. Extended cycles exploit longer wet seasons in the south; shortened cycles escape terminal drought in the north. This is not a description of results but a mechanistic principle derived from systematic parameter exploration.<br>(2) *Source-sink timing versus sink capacity*: The stability of reproductive parameters (G1, G2, G3) across environments, while phenological

	parameters varied substantially, indicates that sink capacity is adequate in current germplasm—the breeding frontier lies in optimizing source-sink timing, not sink strength. This insight redirects breeding priorities from yield components to developmental regulation.<br>(3) *WUE limitations and HI-WUE integration*: Water use efficiency alone can mislead breeding programs: drought-stressed plants with minimal growth may achieve high WUE while producing negligible yield. The HI-WUE composite metric addresses this limitation by rewarding only genotypes combining efficient water use with effective grain formation—excluding "false positives" that conserve water by sacrificing productivity.<br>**Regarding additional traits beyond the current framework:**<br>The model boundaries identified in Discussion (Lines 565–571) explicitly acknowledge traits not captured by CERES-Rice that merit future investigation:<br>• Root architecture: Deeper rooting could access subsoil moisture during terminal drought<br>• Stay-green: Delayed senescence could extend grain filling under moderate stress<br>• Early vigor: Rapid canopy closure could reduce soil evaporation<br>• Osmotic adjustment: Enhanced cellular water retention could maintain turgor under moderate stress<br>These traits represent the "frontier where both [biological and computational expertise] converge—yet this intersection remains largely unexplored" (Line 578).<br>**Changes in manuscript:**<br>• Discussion: Expanded physiological interpretation connecting parameters to mechanisms<br>• Discussion: Added explicit treatment of WUE limitations and HI-WUE

	integration rationale<br>• Discussion: Maintained and strengthened "Modeling living systems: limitations and horizons" section addressing model boundaries and the gap between biological and computational approaches<br>• Conclusion: Added region-specific trait recommendations and future research priorities<br>The manuscript presents not merely a methodological demonstration but a critical framework for advancing process-based modeling beyond routine application toward hypothesis-driven inquiry.

REVIEWER #3

My overall recommendation: Major Revision.

1. Is the manuscript technically sound, and do the data support the conclusions? Partly.

Technically promising, but key methodological gaps (incomplete Morris design specifics, nomulti-seed GA convergence evidence, ad-hoc HI+0.1·WUE objective without robustness/Paretoanalysis, and a problematic similarity normalization) mean the current analyses don't fully support thestrength of the conclusions.

2. Has the statistical analysis been performed appropriately and rigorously? No.

Why:

- Morris sensitivity analysis is under-specified (no numeric k,p,Δ,rk, p, \Delta, rk,p,Δ,r; no design matrixor distribution of elementary effects), so stability/precision can't be assessed.
- GA optimization lacks multi-seed replication and convergence diagnostics; a stochastic optimizerneeds replicate runs with summary stats (mean±SD of best fitness, variability of θ∗\theta^*θ∗).
- The objective J=HI+0.1×WUEJ=\text{HI}+0.1\times\text{WUE}J=HI+0.1×WUE uses an ad-hoc weightwithout scale normalization or sensitivity/Pareto analysis, so results may hinge on the arbitraryα\alphaα.
- The similarity analysis averages heterogeneous distance metrics without standardization and uses anon-monotone normalization (higher similarity → smaller score), with no robustness checks onrankings.
- Uncertainty reporting is limited (few CIs/SDs across sites/years or GA seeds), so inferential strength isunclear.These gaps mean the statistical/analytical treatment isn't yet rigorous enough to support the strengthof the conclusions.

3. Have the authors made all data underlying the findings in their manuscript fully available?. Yes

The manuscript's Data Availability statement points to public repositories (Mendeley Data for datasetsand GitHub for code) with no access restrictions, which satisfies PLOS ONE's policy. As a best-practice enhancement (not a blocker), I recommend archiving a tagged code release with a DOI (e.g., Zenodo) and including per-figure CSVs of the data underlying plotted summaries.

4. Is the manuscript presented in an intelligible fashion and written in standard English? No.
The manuscript is generally understandable, but it does not yet meet PLOS ONE's "clear, correct, unambiguous" English standard. Representative issues that should be corrected at revision:

- Grammar/articles/verb agreement: "a individual point" → an individual point; "the sensitivity analysis assess …" → assesses.
- Typos/word choice: "rainfeed" → rainfed.• Figure captions/labels: duplicated panel label in one figure ("f) Anthesis" should be Maturity); missing spaces around symbols (e.g., "0.50±0.07" → 0.50 ± 0.07).
- Units & style: non-standard units and casing (e.g., Biomass(Kg/H), #/H)—use kg ha^{-1} and no. ha^{-1};WUE shown as kg/mm should be kg ha^{-1} mm^{-1}; LAI listed as mm^2/mm^2 should be m^2 m^{-2} (or "–").
- Notation consistency: parameter code P2O occasionally appears as P20; acronym in a section header shows AG instead of GA.
- Equation formatting/clarity: the "normalized similarity" formula is line-broken and currently maps higher similarity to smaller values—both the typesetting and the monotonicity should be corrected. With a focused language/units pass and caption clean-up, the paper can reach the required standard. ***Please, see attached Letter to the Author and Letter to the Editor as PDF attachment files.***

PLOS ONE evaluates **technical soundness, clarity, ethics/data availability, and reproducibility** rather than perceived impact; my notes, analysis and comments are organized accordingly and include precise locations so you can revise efficiently.

Comment	Answer
3.1. Specify **DSSAT/CERES-Rice versions**, OS, seeds; provide **scripts or a container**.	This specification is appreciated. Software versions and computational environment have been documented:<br>**Computational environment:**<br>• DSSAT v4.8 (CERES-Rice module)<br>• MATLAB R2024b<br>• R v4.4.2<br>• Windows 11 Professional Build 26100.7171<br>• Intel Core i7-12700H processor (12th Gen, 2.30 GHz), 32 GB RAM<br>**Random seeds:** Random seeds for Morris sensitivity analysis and Genetic Algorithm are declared in the code repository. Each analysis uses

<table>
<tr><td></td><td>fixed seeds to ensure reproducibility.
Code availability: All scripts are publicly available at:
GitHub: https://github.com/EdgarStevenC/Crop-Growth-Modelling
Mendeley Data: DOI 10.17632/msg7bfg5t6.1
Changes in manuscript:
Methods (Lines 174–179): Software versions and hardware specifications added
Supplementary Material: Random seed documentation included
Data and Code Availability: Repository links with version information</td></tr>
<tr><td>3.2. Report numeric Morris design parameters (k, p, Δ, r) and attach the design-generation code.</td><td>This specification is appreciated. Morris design parameters have been fully documented:
Morris design parameters:
k = 11 (number of genetic coefficients)
p = 4 (number of levels per parameter)
Δ = parameter-specific step sizes (defined in Figure 4b)
r = 20 (number of trajectories/replications)

Total model evaluations: r × (k + 1) = 20 × 12 = 240 per environment
Design-generation code:
The Morris sampling algorithm and design matrix are included in the GitHub repository:
https://github.com/EdgarStevenC/Crop-Growth-Modelling/morris_analysis/
Changes in manuscript:
Methods: Added explicit Morris parameters (k, p, Δ, r)
Figure 4b: Step sizes (Δ) for each parameter
Table S1: Complete elementary effects distribution (mean ± SD, 95% CI)
Supplementary Material: Morris design matrix and generation code referenced</td></tr>
<tr><td>3.3. Justify HI-WUE weight (0.1) or replace with a Pareto analysis; add a robustness check.</td><td>This concern is acknowledged. The original ad-hoc weighting (α = 0.1) has been replaced with a normalized approach ensuring equal metric</td></tr>
</table>

	contribution.<br>**Revised fitness function:**<br>WUE_norm = (WUE − WUE_min) / (WUE_max − WUE_min) HI-WUE = HI + WUE_norm<br>**Justification:**<br>• HI is intrinsically normalized [0,1] as a biomass ratio (grain yield / total biomass)<br>• WUE is normalized to [0,1] using physiological bounds (2 and 15 kg ha^{-1} mm^{-1}, respectively) established in the literature for drought-adapted aerobic rice<br>• Both metrics contribute equally (50%–50%) to the fitness function<br>This normalization eliminates arbitrary weighting by grounding the scaling in physiological limits reported for the target production system.<br>**Changes in manuscript:**<br>• Methods: Fitness function revised with explicit WUE normalization (Equations 1–2, Supplementary Material)<br>• Methods: Added justification based on literature-derived physiological bounds
3.4. Fix the **similarity normalization** so **higher = more similar**; clarify normalization scope; show stability across metrics.	This concern is acknowledged. The similarity normalization has been revised to ensure monotonic interpretation (higher values = greater similarity).<br>**Similarity formula (Equation 2):**<br>Similarity = 1 − (d̄_ij / max(d̄))<br>where d̄_ij represents the average distance between ideotype i and cultivar j across all metrics, and max(d̄) is the maximum observed distance. This formulation ensures:<br>• Similarity = 1 when distance = 0 (identical)<br>• Similarity = 0 when distance = maximum (most dissimilar)<br>• Monotonic relationship: higher similarity scores indicate greater genetic proximity<br>**Normalization scope:** Distances were normalized globally across all ideotype-cultivar

	pairs to enable cross-environment comparison of breeding candidates.<br>**Stability across metrics:** Ranking stability was assessed by comparing cultivar rankings from individual metrics (Euclidean, Manhattan, Cosine) against the consensus ranking. The top candidates (WAB56-50, DKAP2) remained in the top-3 positions across all individual metrics, confirming robust identification independent of distance measure choice.<br>**Changes in manuscript:**<br>• Methods: Similarity equation with monotonic normalization confirmed (Equation 2)<br>• Methods: Normalization scope (global) explicitly stated<br>• Results: Added statement confirming ranking stability across metrics<br>• Figure 9b: Similarity heatmap with corrected scale (higher = more similar)
3.5. Either **standardize** heterogeneous distance metrics before averaging or justify a single metric (with a robustness check).	The approach of averaging heterogeneous distance metrics has been justified and validated through robustness analysis.<br>**Standardization approach:** Each distance metric was standardized to [0,1] range before averaging:<br>$d\_standardized = (d - d\_min) / (d\_max - d\_min)$<br>This ensures that Euclidean, Manhattan, and Cosine distances contribute equally to the composite similarity score, regardless of their different native scales.<br>**Justification for multiple metrics:** Three complementary metrics capture different aspects of genetic proximity:<br>• Euclidean: geometric distance in parameter space (sensitive to magnitude)<br>• Manhattan: coordinate-wise absolute differences (robust to outliers)<br>• Cosine: angular similarity between coefficient vectors (captures proportional relationships)<br>The consensus across standardized metrics provides more robust cultivar ranking than any single metric alone.

	**Robustness check:** Ranking stability was assessed by comparing: (i) Individual metric rankings vs. consensus ranking (ii) Leave-one-metric-out analysis<br>Results: WAB56-50 and DKAP2 consistently ranked in top-3 positions across all individual metrics and all leave-one-out combinations, with Spearman rank correlation > 0.92 between individual and consensus rankings.<br>**Changes in manuscript:**<br>• Methods: Added explicit standardization procedure for distance metrics<br>• Methods: Justification for multi-metric approach added<br>• Supplementary Material: Robustness analysis with ranking stability statistics
3.6. Provide **GA convergence diagnostics** and **seeded replicates**; briefly assess sensitivity to GA hyperparameters.	Convergence diagnostics and robustness analysis have been added.<br>**Convergence diagnostics:** Fitness trajectories were tracked across 40 generations for each environment. Convergence was defined as fitness improvement < 0.1% over 5 consecutive generations. Results show:<br>-Environment 1: Converged at generation 23<br>-Environment 2: Converged at generation 20<br>-Environment 3: Converged at generation 10<br>-Environment 4: Converged at generation 20<br>**Seeded replicates:** Optimization was repeated with multiple independent random seeds for each environment. Parameter estimates were stable across seeds, with coefficient of variation < 8% for all genetic coefficients, confirming optimization robustness.<br>**Hyperparameter sensitivity:** The GA configuration (population = 15, generations = 40, mutation rate = 0.7) was assessed through sensitivity analysis:<br>• Population size [10, 15, 20]: Optimal solutions consistent across sizes<br>• Mutation rate [0.5, 0.7, 0.9]: High mutation (0.7) facilitated broad search space coverage without premature convergence

	• Generations [20, 30, 40]: Extended generations (40) ensured convergence across all environments<br>***Changes in manuscript:***<br>• Methods: GA hyperparameters explicitly stated with justification<br>• Results: Convergence generation reported for each environment<br>• Figure S1: Convergence trajectories showing fitness stabilization<br>• Table S3: Optimal genetic coefficients with convergence statistics<br>• Supplementary Material: Hyperparameter sensitivity analysis
3.7. Justify (or analyze sensitivity to) **fixed root depth**; move the assumption into Methods.	**Root depth specification:** CERES-Rice uses a fixed soil profile depth of 1 meter, as shown in Figure 8 (Root Deep: 1 m for all environments). This is consistent with DSSAT default configuration and standard agronomic practice for rice root system characterization. This depth encompasses the primary root zone where >90% of water and nutrient uptake occurs in rice.<br>**Justification:**<br>• Rice root systems typically reach 0.6–1.0 m depth under rainfed conditions<br>• The 1-meter profile captures the functional rooting zone relevant for water balance calculations<br>• This parameterization is consistent with field observations in West African rainfed systems and DSSAT protocols for regional-scale applications<br>**Sensitivity consideration:** Root depth is not among the optimized genetic coefficients (P1, P5, P2R, PHINT, P2O, G1, G2, G3) because CERES-Rice treats soil profile as an environmental input rather than a genotypic parameter. The model captures genotypic variation in water uptake through root density coefficients implicitly linked to tillering (G3) and vegetative duration (P1).<br>**Model boundary:** As noted in Discussion (Lines 520–529), root architectural plasticity is not explicitly represented in CERES-Rice—this

<table>
<tr><td></td><td>structural simplification is acknowledged as a model boundary, representing a frontier for future model advancement.
Changes in manuscript:
Figure 8: Root depth (1 m) explicitly shown for all environments
Discussion (Lines 520–529): Root plasticity acknowledged as model boundary</td></tr>
<tr><td>3.8. Implement the English/style fixes enumerated above (units, labels, spelling, spacing, header typos).</td><td>This feedback is appreciated. A comprehensive language and style revision has been implemented:
Notation consistency:
Parameter code "P20" → "P2O" (Line 245)
Placeholder "Figure X" replaced with specific figure number (Line 241)
Units standardization (Figure 8):
Biomass: "Kg/H" → "kg ha^{-1}"
Grain Number/Tillers: "#/m2" → "no. m^{-2}"
WUE: "Kg/mm" → "kg ha^{-1} mm^{-1}"
LAI: "m2/m2" → "m^2 m^{-2}"
Numerical consistency:
GA generations: confirmed as 40 throughout manuscript (Line 237)
Spacing:
Spaces added around symbols where needed (e.g., "±")
Changes in manuscript:
Complete notation and units review throughout
Figure 8: All units standardized
All figure captions reviewed for consistency</td></tr>
<tr><td>3.9. Bring the References into PLOS style (journal names, author spellings/diacritics, punctuation, completeness) and ensure 1-to-1 mapping with in-text citations.</td><td>References have been reviewed and verified against PLOS ONE style guidelines. Journal names, author diacritics, punctuation, and DOI formatting are consistent throughout. All 58 in-text citations map 1-to-1 with the reference list.</td></tr>
<tr><td>3.10. Strengthen Data Availability with a versioned DOI for code.</td><td>This suggestion is appreciated. Data and code availability have been strengthened with versioned DOIs:

Mendeley Data: DOI 10.17632/msg7bfg5t6.1</td></tr>
</table>

	(Version 1, published 2025)<br>Code repository:<br>GitHub: https://github.com/EdgarStevenC/Crop-Growth-Modelling<br>License: Creative Commons Attribution 4.0 International (CC BY 4.0)

AUTHORS' RESPONSE TO JOURNAL REQUIREMENTS

Comments:	Answer
**4.1.** When submitting your revision, we need you to address these additional requirements.<br>Please ensure that your manuscript meets PLOS ONE's style requirements, including those for filenaming. The PLOS ONE style templates can be found at https://journals.plos.org/plosone/s/file?id=wjVg/PLOSOne_formatting_sample_main_body.pdf and https://journals.plos.org/plosone/s/file?id=ba62/PLOSOne_formatting_sample_title_authors_affiliations.pdf	The manuscript has been formatted according to PLOS ONE style templates. File naming conventions have been verified and corrected to meet journal requirements.<br>Changes implemented:<br><br>Manuscript formatted using PLOS ONE LaTeX template<br>File names standardized according to journal guidelines<br>Title, authors, and affiliations formatted per PLOS ONE specifications
**4.2.** In your Methods section, please provide additional information regarding the permits you obtainedfor the work. Please ensure you have included the full name of the authority that approved the fieldsite access and, if no permits were required, a brief statement explaining why.	This study used publicly available datasets from previously published field experiments. No new field sampling was conducted, and therefore no permits or ethical approvals were required. Soil data were derived from SoilGrids, climate data from NASA POWER, and crop observations from Gérardeaux et al. (2021), who obtained all necessary permits for the original data collection.<br>**Changes in manuscript:**<br>• Data and Code Availability section (Line 622): Statement added clarifying that no permits were required
**4.3.** Please update your submission to use the PLOS LaTeX template. The template and more informationon our requirements for LaTeX submissions can be found at http://journals.plos.org/plosone/s/latex	The manuscript has been formatted using the official PLOS ONE LaTeX template as specified in the journal guidelines.
**4.4.** We note that the grant information you provided in the 'Funding Information' and 'FinancialDisclosure' sections do not match.	The funding information has been reviewed and harmonized across all sections.<br>**Funding sources:**

When you resubmit, please ensure that you provide the correct grant numbers for the awards youreceived for your study in the 'Funding Information' section.	• Agropolis Fondation: CropModAdapt project (Contract No. 2201-026, 2023–2024)<br>• ClimBeR initiative – France–CGIAR Action Plan on Climate Change (ICARDA Agreement No. 200303, 2023–2024)<br>**Financial Disclosure statement:** "This research was funded by the Agropolis Fondation through the CropModAdapt project (Contract No. 2201-026, 2023–2024), and by the ClimBeR initiative – France–CGIAR Action Plan on Climate Change (ICARDA Agreement No. 200303, 2023–2024). The funders had no role in study design, data analysis, decision to publish, or preparation of the manuscript."<br>**Changes in manuscript:**<br>• Funding section (Lines 614–616): Verified consistency with Financial Disclosure<br>• Funding Information and Financial Disclosure sections: Now identical
**4.5.** Please know it is PLOS ONE policy for corresponding authors to declare, on behalf of all authors, allpotential competing interests for the purposes of transparency. PLOS defines a competing interest asanything that interferes with, or could reasonably be perceived as interfering with, the full andobjective presentation, peer review, editorial decision-making, or publication of research or non-research articles submitted to one of the journals. Competing interests can be financial or non-financial, professional, or personal. Competing interests can arise in relationship to an organization oranother person. Please follow this link to our website for more details on competing interests: http://journals.plos.org/plosone/s/competing-interests.	**Competing Interests statement:**<br>*"The author has read the journal's policy on competing interests and, for the purposes of transparency, declares the following. No personal financial compensation beyond the funding sources listed in the Funding section has been received.*<br><br>*The following individuals have potential financial or intellectual property interests related to this work and associated professional relationships that could be perceived as influencing objectivity during the peer review process:*<br><br>*María Camila Rebolledo contributed to the early conceptualization of the project's needs and provided guidance on potential applications, and may have interests in intellectual property arising from this research.*<br><br>*Julian Ramirez-Villegas and Alexandre B. Heinemann have professional or personal connections to the funding or intellectual property associated with this research.*

	The following individuals had prior mentoring relationships with the author that could be perceived as a potential influence:<br><br>*Myriam Adam and Julian Colorado, as previous mentors, provided mentorship and collaborative support during formative stages of this research, which could be perceived as potentially influencing aspects of the research presentation of the results.*<br><br>*The author affirms sole authorship of this work, conducted within the framework of his doctoral research. All other individuals declare no competing interests."*
**4.6.** Thank you for uploading your study's underlying data set. Unfortunately, the repository you havenoted in your Data Availability statement does not qualify as an acceptable data repository accordingto PLOS's standards.<br>At this time, please upload the minimal data set necessary to replicate your study's findings to astable, public repository (such as figshare or Dryad) and provide us with the relevant URLs, DOIs, oraccession numbers that may be used to access these data. For a list of recommended repositories andadditional information on PLOS standards for data deposition, please see https://journals.plos.org/plosone/s/recommended-repositories.	The dataset and code have been uploaded to Zenodo, a PLOS-recommended repository.<br>Data and Code repository:<br><br>Zenodo: DOI 10.5281/zenodo.18094654<br>URL: https://doi.org/10.5281/zenodo.18094654<br>GitHub: https://github.com/EdgarStevenC/Crop-Growth-Modelling<br>License: CC BY 4.0<br><br>Repository contents:<br><br>Sensitivity analysis results (20 replications)<br>Genetic algorithm optimization outputs (4 environments, 40 generations)<br>Similarity analysis data (21 field-validated cultivars)<br>All code to replicate figures and tables<br><br>Changes in manuscript:<br><br>Data and Code Availability section: Updated with Zenodo DOI
**4.7.** We note that Figure 1 in your submission contain copyrighted images. All PLOS content is publishedunder the Creative Commons Attribution License (CC BY 4.0), which means	**Figure 1:** Original conceptual diagram created by the author using AI-assisted image generation tools (DALL-E). No copyrighted images from third parties included. Licensed under CC BY 4.0.

that the manuscript, images, and Supporting Information files will be freely available online, and any third party ispermitted to access, download, copy, distribute, and use these materials in any way, evencommercially, with proper attribution. For more information, see our copyright guidelines: http://journals.plos.org/plosone/s/licenses-and-copyright.<br><br>We require you to either present written permission from the copyright holder to publish these figuresspecifically under the CC BY 4.0 license, or remove the figures from your submission:<br>**a.** You may seek permission from the original copyright holder of Figure 1 to publish the contentspecifically under the CC BY 4.0 license.<br>We recommend that you contact the original copyright holder with the Content Permission Form(http://journals.plos.org/plosone/s/file?id=7c09/content-permission-form.pdf) and the following text:<br>"I request permission for the open-access journal PLOS ONE to publish XXX under the CreativeCommons Attribution License (CCAL) CC BY 4.0 (http://creativecommons.org/licenses/by/4.0/). Pleasebe aware that this license allows unrestricted use and distribution, even commercially, by third parties.Please reply and provide explicit written permission to publish XXX under a CC BY license andcomplete the attached form."<br>Please upload the completed Content Permission Form or other proof of granted permissions as an"Other" file with your submission.<br>In the figure caption of the copyrighted figure, please include the following text: "Reprinted from [ref]under a CC BY license, with permission from [name of publisher], original	**Figure 2:** Original diagram created by the author using AI-assisted tools. Three microscopy images are included for illustration from open-access publications, all under CC BY license.<br>***Permission Declaration for Figure 2:***<br>The author confirms that all microscopy images used in Figure 4 are already published under a Creative Commons Attribution license (CC BY) as follows:<br><br>1. Fan et al. (2023), Frontiers in Plant Science, DOI: 10.3389/fpls.2023.1074148.<br>Copyright © 2023 Fan, Xu, Wang, Liu, Zhang, Tian, Wei and Zhang. This is an open-access article distributed under the terms of the CC BY license.<br>• Fan et al. (2023), *Frontiers in Plant Science* (CC BY):<br>https://doi.org/10.3389/fpls.2023.1074148<br><br>2. Liu et al. (2016), Frontiers in Plant Science, DOI: 10.3389/fpls.2016.01006.<br>Copyright © 2016 Liu, Li, Su, Li and Shi. Distributed under the CC BY license.<br>• Liu et al. (2016), *Frontiers in Plant Science* (CC BY):<br>https://doi.org/10.3389/fpls.2016.01006<br><br>3. Yang et al. (2025), Plant Methods, DOI: 10.1186/s13007-025-01451-z.<br>Licensed under the CC BY 4.0 license.<br>• Yang et al. (2025), *Plant Methods* (CC BY):<br>https://doi.org/10.1186/s13007-025-01451-z<br><br>These images are freely available under CC BY for use, distribution, and reproduction with proper credit. Therefore, no additional permissions from the copyright holders are required.<br><br>The caption in the figure include "Source: Original diagram created by the author using AI-assisted tools. Microscopy images used for illustration are reprinted from Fan et al. (2023) (31), Liu et al.

copyright [original copyrightyear].”<br>**b.** If you are unable to obtain permission from the original copyright holder to publish these figuresunder the CC BY 4.0 license or if the copyright holder’s requirements are incompatible with the CC BY4.0 license, please either i) remove the figure or ii) supply a replacement figure that complies with theCC BY 4.0 license. Please check copyright information on all replacement figures and update the figurecaption with source information. If applicable, please specify in the figure caption text when a figure issimilar but not identical to the original image and is therefore for illustrative purposes only.	(2016) (32), and Yang et al. (2025) (33) under a CC BY license, with permission from the respective publishers, original copyright 2023, 2016, and 2025. All files are freely available online for use, distribution, and reproduction with proper attribution”
**4.8.** We note that Figure 2 in your submission contain map images which may be copyrighted. All PLOScontent is published under the Creative Commons Attribution License (CC BY 4.0), which means thatthe manuscript, images, and Supporting Information files will be freely available online, and any thirdparty is permitted to access, download, copy, distribute, and use these materials in any way, evencommercially, with proper attribution. For these reasons, we cannot publish previously copyrightedmaps or satellite images created using proprietary data, such as Google software (Google Maps, Street View, and Earth). For more information, see our copyright guidelines: http://journals.plos.org/plosone/s/licenses-and-copyright.<br>We require you to either present written permission from the copyright holder to publish these figuresspecifically under the CC BY 4.0 license, or remove the figures from your submission:<br>**a.** You may seek permission from the original copyright holder of Figure 2 to publish the contentspecifically under the CC BY 4.0 license.<br>We recommend that you contact the original	Figure 2 (now Figure 3 in the revised manuscript) was generated by the author using MATLAB R2024b with Mapping Toolbox and Image Processing Toolbox. The figure is adapted from Correa et al. (2025), published under CC BY license in Discover Food (Springer Nature). As the original author, full rights are retained to reuse and adapt this figure. No proprietary map sources (Google Maps, etc.) were used.<br>**Changes in manuscript:**<br>• Figure 3 caption: Added "Source: generated using MATLAB R2024b with Mapping Toolbox and Image Processing Toolbox (https://www.mathworks.com)"

copyright holder with the Content Permission Form(http://journals.plos.org/plosone/s/file?id=7c09/content-permission-form.pdf) and the following text:<br>“I request permission for the open-access journal PLOS ONE to publish XXX under the CreativeCommons Attribution License (CCAL) CC BY 4.0 (http://creativecommons.org/licenses/by/4.0/). Pleasebe aware that this license allows unrestricted use and distribution, even commercially, by third parties.Please reply and provide explicit written permission to publish XXX under a CC BY license andcomplete the attached form.”<br>Please upload the completed Content Permission Form or other proof of granted permissions as an"Other" file with your submission.<br>In the figure caption of the copyrighted figure, please include the following text: “Reprinted from [ref]under a CC BY license, with permission from [name of publisher], original copyright [original copyrightyear].”<br>**b.** If you are unable to obtain permission from the original copyright holder to publish these figuresunder the CC BY 4.0 license or if the copyright holder’s requirements are incompatible with the CC BY4.0 license, please either i) remove the figure or ii) supply a replacement figure that complies with theCC BY 4.0 license. Please check copyright information on all replacement figures and update the figurecaption with source information. If applicable, please specify in the figure caption text when a figure issimilar but not identical to the original image and is therefore for illustrative purposes only.<br>The following resources for replacing copyrighted map figures may be helpful:<br>USGS National Map Viewer (public domain): http://viewer.nationalmap.gov/viewer/<br>The Gateway to Astronaut Photography of	

Earth (public domain): http://eol.jsc.nasa.gov/sseop/clickmap/<br>Maps at the CIA (public domain): https://www.cia.gov/library/publications/the-world-factbook/index.html and https://www.cia.gov/library/publications/cia-maps-publications/index.html<br>NASA Earth Observatory (public domain): http://earthobservatory.nasa.gov/<br>Landsat: http://landsat.visibleearth.nasa.gov/<br>USGS EROS (Earth Resources Observatory and Science (EROS) Center) (public domain): http://eros.usgs.gov/#<br>Natural Earth (public domain): http://www.naturalearthdata.com/	
**4.9.** If the reviewer comments include a recommendation to cite specific previously published works,please review and evaluate these publications to determine whether they are relevant and should becited. There is no requirement to cite these works unless the editor has indicated otherwise.	The reviewer comments have been reviewed. No specific citation recommendations were made by the reviewers. All citations in the manuscript were selected based on their direct relevance to the methodology and findings presented.

Sincerely,

Edgar S. Correa.
Pontificia Universidad Javeriana, Date: 29/12/2025
