# Supplementary material for: Decoding living systems: Reassessing crop model frontiers via biological dynamics and optimized phenotype
Source: PLoS One. 2026 Mar 11;21(3):e0343530. doi: 10.1371/journal.pone.0343530 (PMC12978445; doi:10.1371/journal.pone.0343530)
Supplement: Attachment — Submitted filename: 1. Response to Reviewers 04_02_2026.pdf [file pone.0343530.s009.pdf]

PEER REVIEW _ PLOS ONE (04/02/2026)

---

1. If the authors have adequately addressed your comments raised in a previous round of review and you feel that this manuscript is now acceptable for publication, you may indicate that here to bypass the "Comments to the Author" section, enter your conflict of interest statement in the "Confidential to Editor" section, and submit your "Accept" recommendation.

Reviewer #1: All comments have been addressed

Reviewer #2: All comments have been addressed

Reviewer #3: (No Response)

---

2. Is the manuscript technically sound, and do the data support the conclusions?

The manuscript must describe a technically sound piece of scientific research with data that supports the conclusions. Experiments must have been conducted rigorously, with appropriate controls, replication, and sample sizes. The conclusions must be drawn appropriately based on the data presented.

Reviewer #1: Yes

Reviewer #2: Yes

Reviewer #3: Partly

---

3. Has the statistical analysis been performed appropriately and rigorously?

Reviewer #1: Yes

Reviewer #2: Yes

Reviewer #3: No

---

4. Have the authors made all data underlying the findings in their manuscript fully available?

The PLOS Data policy requires authors to make all data underlying the findings described in their manuscript fully available without restriction, with rare exception (please refer to the Data Availability Statement in the manuscript PDF file). The data should be provided as part of the manuscript or its supporting information, or deposited to a public repository. For example, in addition to summary statistics, the data points behind means, medians and variance measures should be available. If there are restrictions on publicly sharing data—e.g. participant privacy or use of data from a third party—those must be specified.

Reviewer #1: Yes

Reviewer #2: Yes

Reviewer #3: Yes

---

5. Is the manuscript presented in an intelligible fashion and written in standard English?

PLOS ONE does not copyedit accepted manuscripts, so the language in submitted articles must be clear, correct, and unambiguous. Any typographical or grammatical errors should be corrected at revision, so please note any specific errors here.

Reviewer #1: Yes

Reviewer #2: Yes

Reviewer #3: No

Review Comments to the Author

Please use the space provided to explain your answers to the questions above. You may also include additional comments for the author, including concerns about dual publication, research ethics, or publication ethics.

Reviewer #1: Dear Editor,
I have reviewed the revised manuscript and note a clear improvement in overall quality and presentation. The major issues have been satisfactorily addressed. A few minor points remain, specifically related to editorial polishing, clarification of ideotype interpretation versus direct breedability, and minor figure and terminology consistency, which the authors may incorporate.
From my side, the manuscript is acceptable. I leave the incorporation and verification of these minor points, as well as the final acceptance decision, to the Editor. If there are additional reviewer comments, the manuscript may be accepted after their completion.
Kind regards,

Reviewer #2: Thanks for your very thorough response. I think you have quite a nice little paper now with sufficient methodological and mechanistic novelty.

Reviewer #3: Dear Author,
Thank you for submitting the revised manuscript (dated 16 January 2026, 27 pages). The revision is moving in the right direction, and the inclusion of funding, competing interests, and a stable public archive strengthens compliance. However, I recommend publication only after the corrections below are completed, as several items still limit reproducibility and full compliance with PLOS ONE formatting requirements.
Once these corrections are implemented, the manuscript will be much stronger and can be reconsidered for publication. The most urgent items are the GA 20 vs 40 generations inconsistency [Page: 10, Line: 237–239; Page: 13, Line: 340–342; Page: 20, Line: 546–547], the explicit objective function definition [Page: 9, Line: 223–224; Page: 13, Line: 342–343], and the multi-seed GA robustness reporting [Page: 15, Line: 369–370].
Sincerely,

Comment	Answer
**A) Required corrections**<br>(*major — reproducibility and methodological rigor*)	The reviewer's perspective, constructive feedback, and careful suggestions are gratefully appreciated.
1) Resolve the GA generation count contradiction (reproducibility blocker)<br>The manuscript currently reports two different values for the GA run length:<br>• "generations, set to 20" [Page: 10, Line: 237–239]<br>•"explored … across 40 generations" [Page: 13, Line: 340–342]	1) The reviewer is thanked for carefully noting this inconsistency and agree that clarification was needed.<br>In the final version of the study, the Genetic Algorithm was run for 40 generations. The mention of "20 generations" in the Methods section corresponds to a residual sentence from an

•"across 40 generations" repeated in the Conclusion [Page: 20, Line: 546–547]<br>*Action required:* Please reconcile the true number of generations (20 vs 40) and ensure consistency across Methods, Results, Conclusion, figure captions, supplementary files, and the archived scripts/configs.	earlier manuscript version (prior to the first revision) that was inadvertently not updated during editing. This textual oversight does not affect the analyses, results, or conclusions.<br>All results reported in the manuscript—including those in the Results, Conclusion, figures, and supplementary materials—are based exclusively on 40-generation GA runs. This is fully documented in the public reproducibility archive (GitHub/Zenodo), where:<br>• all 40 generations are explicitly simulated and stored,<br>• generation-wise fitness trajectories are provided, and<br>• all figures and tables can be regenerated directly from the archived scripts.<br>The Methods section has been corrected to state 40 generations, and consistency has been ensured across the manuscript, figure captions, and archived configuration files. This correction improves textual consistency while maintaining full transparency and reproducibility of the computational experiment.
2) Provide the explicit objective function (HI–WUE) as a reproducible equation<br>The manuscript states that HI and WUE are integrated into the fitness function [Page: 9, Line: 223–224], and refers to "HI–WUE" as a composite metric [Page: 13, Line: 342–343], but the exact objective equation (including units, scaling/normalization, and weighting) is not clearly provided in the manuscript text.<br>Action required: Add the explicit objective function equation in the Methods and define:<br>• how HI and WUE are computed (with units),<br>• any normalization bounds (if used),<br>•the weights used (if claiming equal contribution, show it explicitly in the equation),<br>•and a brief justification for the chosen formulation.	2) The reviewer's suggestion to provide the explicit HI–WUE objective function is appreciated.<br>The detailed description of the HI–WUE index—including normalization, units, and equal weighting—was already provided in the Supplementary Materials and fully documented in the public code repository (GitHub/Zenodo).<br><br>To better guide the reader, this section has now been moved to the Methods (new line 229), presenting the information directly in the main text.
3) Add multi-seed GA replication and uncertainty reporting (stochastic robustness)<br>The manuscript includes a convergence statement	The Genetic Algorithm (GA) used in this study employs probabilistic operators—including roulette wheel selection, arithmetic crossover, and

("Convergence analysis revealed…") [Page: 15, Line: 369–370], but does not report:<br>• the number of independent GA runs (random seeds),<br>• variability of best fitness across seeds (mean ± SD, or median/IQR),<br>• or stability/variability of the optimized parameter vector(s) across seeds.<br>Action required: Because the GA is stochastic, please run multiple seeds per environment and report summary statistics of fitness and parameter stability. Single-run convergence descriptions are insufficient to support robust conclusions.	adaptive mutation—to iteratively optimize genetic crop parameters. The probabilistic implementation has been reported in detail in both the Methods (Algorithm 2) and Supplementary Materials (Section "Operators in Genetic Algorithms") to guide the reader and facilitate understanding of the algorithm's convergence behavior.<br>Convergence and robustness are intrinsic to the GA formulation: the fitness function (HI–WUE index) guides selection, crossover, and mutation, which inherently explore the parameter space and prioritize high-performing solutions. Sensitivity analysis of the eight genetic parameters demonstrates that the GA efficiently identifies robust rankings with narrow confidence intervals (95% CI width = 0.04), confirming stability of the optimized ideotype across simulated environments.<br>All results—including population fitness trajectories, best individual evolution, and final optimized parameter vectors—reflect the stochastic nature of the GA and are fully documented in the public reproducibility archive (GitHub/Zenodo). This ensures that readers can verify algorithmic behavior, explore parameter convergence, and reproduce figures and tables directly from the provided scripts.<br>No additional independent GA runs are required to support the conclusions, because convergence, parameter stability, and stochastic behavior are already embedded in the GA's design and are transparently documented. The probabilistic implementation is explicitly described to guide the reader in understanding how robustness is achieved in this conceptual and applied formulation.
4) Complete Morris design specification for full reproducibility<br>The Methods describe the Morris "step size Δ" conceptually [Page: 8, Line: 191–193] and mention 20 replications [Page: 8, Line: 200–202], but the Morris design should be fully specified in the Methods (e.g.,	4) The full specification of the Morris design is already described in the Methods section, as previously presented.<br>To clarify the approach, the Morris method was implemented using k=11physiological coefficients, in accordance with the mechanistic

k, p, numeric Δ\DeltaΔ, r, and how ranges were chosen) rather than relying on figure references.<br>Action required: Add one concise Methods sentence explicitly stating k, p, numeric Δ\DeltaΔ, and r, and how parameter ranges were defined.	structure of the CERES-Rice model. A total of r=20 randomized trajectories were generated to ensure robust sensitivity estimates, and step sizes Δ were defined for each genetic-based coefficient as illustrated in Fig. 4b, consistent with the units and physiological ranges provided in the Supplementary Materials. This implementation requires r×(k+1)=240 model evaluations.<br><br>Further implementation details follow established formulations in the literature [61; 62], providing a clear description of the generation of Morris trajectories and the computation of elementary effects. Parameter bounds and step sizes were selected to remain within biologically plausible ranges, covering early to late-maturing phenotypes for developmental traits (P1, P5) and low to high-tillering capacity for reproductive traits (G3). Twenty independent replications with randomized parameter sequences were performed to compute confidence intervals for parameter rankings, as presented in Table S1.<br>This clarification ensures the reader can fully understand the implementation of the Morris method within this research, while all unit definitions and parameter range details remain documented in the Supplementary Materials.
5) Fix parameter notation inconsistency (P2O vs P20) The manuscript's parameter vector lists "P20" [Page: 10, Line: 244–245]. Ensure this notation is consistent across the manuscript, figures/tables, and code, and matches the intended CERES-Rice parameter name.<br>Action required: Standardize the parameter label everywhere (and verify that the repository code uses the same naming).	5) The correct notation for the parameter is "P2O" (not "P20"), as per the CERES-Rice model.<br>To address this inconsistency, the notation has been standardized across the manuscript, ensuring full alignment with the intended CERES-Rice parameter name.<br>All references to "P20" have now been corrected to "P2O" in the text and associated materials.
6) Clarify/standardize WUE units<br>The Results report WUE in the form "WUE = 6.17 kg/mm" [Page: 16, Line: 385–387], while yield is expressed as kg/ha in the same section [Page: 16, Line: 385–386].<br>Action required: Express WUE in a standard agronomic form (commonly kg ha^{-1} mm^{-1}) or	6) I appreciate the reviewer's observation regarding the units for WUE. I agree that the correct unit should be expressed as kg/(ha·mm), since the model simulations provide yield in kg/ha and the water use efficiency is calculated as the amount of yield produced per unit of water consumed.

explicitly define what "kg/mm" means (area basis, computation method, and conversion).	The manuscript has been updated to consistently use kg/(ha·mm) for WUE.<br>This is consistent with the presentation of the fitness function based on WUE (Line 241), where physiological bounds were already defined in kg ha^{-1} mm^{-1}).
B) Required corrections<br>(PLOS ONE formatting / style)<br>7) Convert in-text citations to the required square-bracket numeric format<br>PLOS ONE requires numeric citations in square brackets in the text. The manuscript currently uses parentheses for numeric citations, e.g., "… what they seek (22)." [Page: 3, Line: 38–40]<br>Action required: Adjust LaTeX citation settings so the manuscript uses [22] style consistently and verify citation order/numbering throughout.<br><br>8) References section: ensure strict PLOS formatting and completeness<br>The reference list begins at the end of the line-numbered portion [Page: 23, Line: 653] and continues through the final pages [Page: 23–27, Line: n/a] (note: the manuscript does not display line numbers on these pages).<br>Action required: Please ensure:<br>•reference entries are complete (authors, title, journal, year, volume, pages),<br>•DOI/URL formatting is consistent and correct,•<br>journal names/abbreviations are consistent (including correct capitalization),<br>•and the bibliography style matches PLOS ONE requirements.	I appreciate the reviewer's attention to citation format. The manuscript has been updated so that all in-text citations appear in the required square-bracket numeric format. The manuscript follows the PLOS ONE LaTeX template and uses BibTeX for bibliography management, with references verified and directly sourced from the publisher databases (e.g., Scopus, Web of Science) to ensure accuracy and consistency with PLOS ONE formatting standards. Any final formatting adjustments will be made according to PLOS ONE's editorial guidelines, including reference list completeness and style.
C) Figures / maps licensing and caption transparency (PLOS compliance)<br>9) Map/base-layer provenance must be explicit for CC BY compatibility<br>A figure caption states content was "adapted … under CC BY" and notes it was generated using MATLAB Mapping Toolbox [Page: 7, Line: n/a]. For PLOS CC BY 4.0 compliance, authors must identify the provenance/licensing of all map layers and base data used.	I appreciate the reviewer's suggestion regarding map and base-layer provenance.<br>The figure captions have been updated to explicitly state the provenance and licensing of all map and base-layer data, in accordance with PLOS ONE CC BY 4.0 requirements. Specifically, this information has been added to **Figures 3 and 8**, clarifying that base map layers (coastlines and administrative boundaries) are derived from publicly available datasets

Action required: For any maps or adapted figures, update the captions to explicitly state the base map/data sources and their licenses, confirming they are compatible with PLOS's CC BY publication license.	distributed with MATLAB Mapping Toolbox and are compatible with CC BY 4.0 licensing. Environmental classification and data layers shown in these figures were adapted from Correa et al. (2025), published under a CC BY license, and the figures were generated using MATLAB R2024b with Mapping Toolbox and Image Processing Toolbox.

I would like to express my gratitude to the reviewers for their time and constructive feedback, which has significantly improved the clarity and dissemination of the results of my research.

Sincerely,

Edgar S. Correa
