# Supplementary material for: Decoding living systems: Reassessing crop model frontiers via biological dynamics and optimized phenotype
Source: PLoS One. 2026 Mar 11;21(3):e0343530. doi: 10.1371/journal.pone.0343530 (PMC12978445; doi:10.1371/journal.pone.0343530)
Supplement: Attachment — Submitted filename: Review Report.docx [file pone.0343530.s005.docx]

**Review Report**

**Title:** **Mechanistic crop modelling and AI for ideotype optimization: Crop-scale advances to enhance yield and water use efficiency**
The manuscript presents a novel integration of mechanistic crop modeling (CERES-Rice) and AI (Genetic Algorithm) for ideotype optimization. It is methodologically sound, well-organized, and offers valuable insights for precision agriculture.

**Major Comments with Line References**

1. Line 51–56: Justify the selection of 1,884 virtual cultivars and 5,692 runs; clarify stopping criteria and convergence.
2. Lines 208–215: Genetic Algorithm parameters (population size = 15, generations = 20) seem low; justify or consider alternatives.
3. Algorithm 2: No mention of overfitting control; suggest cross-validation or bootstrapping.
4. Line 52 & 417: Number of environments (n=4) may limit generalizability; validate across more diverse settings.
5. Fig. 6 & Lines 327–366: Add confidence intervals or significance testing to support sensitivity rankings.
6. Fig. 7 & 8: Report statistical significance of correlation results.
7. Line 423–426: Promising cultivar claims based on similarity metrics alone; field validation needed.
8. Line 457: Present PCA variance (e.g., 86.05%) in tabular form.
9. Lines 1–60 (Abstract): Too dense; trim to emphasize key findings and novelty.
10. Lines 545–560 (Conclusion): Avoid repetition; stress broader implications for breeding.
11. Line 376–393: Break long paragraphs for better readability.
12. Lines 20–40: Include foundational references (e.g., Donald’s ideotype concept).
13. Line 68–87: Avoid excessive reuse of [32]; clarify citation purpose.
14. Line ~500: Ensure ethics statement is clearly included in manuscript.
15. Line 536–541: Briefly discuss potential future ethical considerations.

**General Notes**

16. Ensure all figures (e.g., 7 & 8) are fully labeled with units.
17. Include summary statistics (e.g., mean ± SD) in figure captions.
18. Add version numbers of DSSAT, Python packages, etc.
19. Include a reproducibility checklist or flowchart as supplementary material.

**Final Recommendation: Major Revision**

The study is innovative and promising but requires additional methodological justification, statistical clarity, and formatting refinements before acceptance.
